# Striking efficacy of a vaccine targeting TOP2A for triple-negative breast cancer immunoprevention

**DOI:** 10.1038/s41698-023-00461-1

**Published:** 2023-10-25

**Authors:** Sang Beom Lee, Jing Pan, Donghai Xiong, Katie Palen, Bryon Johnson, Ronald A. Lubet, Robert H. Shoemaker, Jeffrey E. Green, Romaine Ingrid Fernando, Shizuko Sei, Ming You, Yian Wang

**Affiliations:** 1 Center for Cancer Prevention, Houston Methodist Cancer Center, Houston Methodist Research Institute, Weill Cornell College of Medicine, Houston, TX USA; 2 Division of Hematology and Oncology, Department of Medicine, Medical College of Wisconsin, Milwaukee, WI USA; 3 Chemopreventive Agent Development Research Group, Division of Cancer Prevention, National Cancer Institute, Bethesda, MD USA; 4 Laboratory of Cancer Biology and Genetics, National Cancer Institute, NIH, Bethesda, MD USA

**Keywords:** Cancer genetics, Cancer

## Abstract

Triple-negative breast cancer (TNBC) is an aggressive subtype of breast cancer that has a poor prognosis. TOP2A is a key enzyme in DNA replication and is a therapeutic target for breast and other cancers. TOP2A-specific Th1-promoting epitopes with optimal binding affinity to MHC II were identified using a combined scoring system. The multi-peptide TOP2A vaccine elicited a robust immunologic response in immunized mice, as demonstrated by the significant production of Th1 cytokines from immunized animals’ splenocytes stimulated in vitro with TOP2A peptides. Anti-tumor efficacy of the TOP2A vaccine was demonstrated in a syngeneic TNBC mouse model, in which pre-graft preventive vaccination was associated with significantly decreased tumor growth as compared to adjuvant control. In a genetically engineered mouse (GEM) model of TNBC, vaccinated animals demonstrated a significant reduction in tumor incidence and average tumor volume compared to adjuvant control. Finally, we examined TCR sequences in CD4 tumor Infiltrating lymphocytes (TIL) from vaccinated mice and found that the TIL contained TCR sequences specific to the three vaccine peptides. These data indicate that our newly developed multi-peptide TOP2A vaccine is highly immunogenic, elicits TILs with vaccine specific TCRs, and is highly effective in preventing and intercepting TNBC development and progression in vivo.

## Introduction

Breast cancer is the leading malignancy in women with 281,550 estimated new cases and 43,600 estimated deaths reported in 2023 in the United States^1^. Approximately 20% of breast cancers are classified as triple-negative breast cancer (TNBC) as they do not express estrogen receptor (ER), progesterone receptor (PR) and human epidermal growth factor receptor 2 (HER2), encompassing the molecular subtypes of basal-like and the more recently defined claudin-low group^2^. TNBC is the subtype of breast cancer observed in women with BRCA-1 germline mutations. In the sporadic setting, TNBC is also associated with African Americans, younger age, higher grade and mitotic index, and more advanced stage at diagnosis^3^. Specific risk factors that correlate with TNBC include reproductive factors (pregnancy and multiple childbirths) and obesity. In TNBC patients, the 5-year survival rate is much lower than other types of breast cancer including ER^+^PR^+^HER2^−^ (Luminal A) and HER2^+^ subtypes^4,5^. Luminal A tumors have highly effective targeted therapies, e.g. hormonal agents (tamoxifen, aromatase inhibitors), whereas HER2^+^ breast cancers are treated with anti-HER2 antibodies or small molecule HER2-kinase inhibitors^6^. In contrast, early and intermediate stages of TNBC are routinely treated with standard cytotoxic chemotherapy resulting in strong initial regression in most patients; however, resistance to chemotherapy subsequently develops in most patients. With the current challenges in treating TNBC, new approaches are needed to improve clinical outcomes.

Cancer vaccines studied to date have primarily been examined in late-stage cancers, which provide limited efficacy most likely due to the immune suppressive tumor microenvironment intrinsic to advanced malignancies. Immune suppression can involve multiple mediators including T regulatory cells (Treg), inhibitory macrophages and other suppressive factors. One way to potentially use cancer vaccines more effectively is in the prevention or early interception setting. If the antigens are expressed early in oncogenesis, vaccines could have utility in prevention. Several reports have now provided proof-of-concept to support the use of peptide vaccines targeting overexpressed self-antigens for cancer immunoprevention^7–10^. Molecular analysis of tumors has identified many genes that are overexpressed in breast cancer, which can be exploited as tumor antigens and potential vaccine candidates. Currently, the most common tumor antigens used in cancer immunotherapy are upregulated self-proteins, such as HER2. Vaccination with peptides targeting overexpressed HER2 in humans has been shown to be effective and well-tolerated^11,12^. While mutated epitopes are recognized as foreign “neo-antigens” by the immune system, eliciting a type 1 immune response, epitopes derived from non-mutated self-antigens are more likely to trigger T helper 2 (Th2) cytokines such as interleukin (IL)-10 and IL-4 that can inhibit cytotoxic T-lymphocyte (CTL) proliferation and function. Recently, attempts have been made to specifically identify Th1-selective epitopes from non-mutated self-antigens that can elicit a Th1 response, when used in a vaccine, can elicit unopposed type 1 immunity and can be effective in preventing cancer growth in preclinical models. If Th2-inducing epitopes from the same protein are included in a vaccine, Th2 cells elicited by immunization may abrogate the Th1-mediated anti-tumor effect and therefore, it is imperative to remove Th2 epitopes from cancer vaccines^12,13^.

In the current study, we used The Cancer Genome Atlas (TCGA) databases and transcriptomic and/or proteomic analysis of normal vs. malignant human breast tissues to identify highly expressed genes in the malignant tissues. We found that topoisomerase 2 alpha (TOP2A) is highly expressed in human TNBC (Supplemental Fig. 1). TOP2A is a known key enzyme in DNA replication, cancer cell proliferation and a direct or indirect target of several cytotoxic anticancer agents (e.g. anthracyclines and etoposides). Recent studies suggested that TOP2A has potential applications in breast cancer detection and management^14^. We constructed a TOP2A multi-peptide vaccine and evaluated its immunogenicity and preventive efficacy against TNBC in the C3(1)/Tag mouse model, which has been utilized extensively because of its genetic similarity to the human basal subtype of TNBC^15,16^. Both C3(1)/Tag TNBCs and mammary carcinoma cell lines (derived from C3(1)/Tag mammary tumors) implanted into the mammary fat pads have been reported to spontaneously metastasize to the lung and liver^17^. By using single-cell RNA sequencing (scRNAseq) analyses we show that TOP2A multi-peptide vaccination induces anti-tumor CD4 + Th1 cells and cytotoxic CD8 + T cells in mouse breast tumor and lymph node tissue samples. Additionally, we have found that the TOP2A vaccine induces a potent TOP2A-specific memory immune response that rejected secondary tumor challenge. Finally, we examined TCR sequences from CD4 tumor infiltrating lymphocytes (TIL) in tumors from vaccinated mice and detected TILs with TCR sequences against all the immunizing peptides. Taken together, our data demonstrate that the multi-peptide TOP2A vaccine is highly immunogenic and efficacious in the prevention of TNBC and warrants further investigation.

## Results

### Gene expression analyses of TOP2A

We found that the TOP2A gene was overexpressed in various mouse breast tumor cell lines and human TNBC and lung tumors (Supplemental Fig. 1). In mice, the TOP2A gene was overexpressed in mouse mammary tumor cell lines M27 (mammary benign tumor cell line), M6 (mammary malignant tumor cell line), and M6C (mammary metastatic tumor cell line), with the M28 mammary cell line serving as a normal control (Supplemental Fig. 1a). The *TOP2A* gene was also highly overexpressed in TCGA TNBC samples (12 normal breast tissues and 137 tumor samples of the TNBC patients) with limited expression in normal mammary tissues (Supplemental Fig. 1b). We also performed expression analysis of TNBC in African Americans (AA), who are at high risk for TNBC. Similar to the general TNBC population, *TOP2A* was significantly overexpressed in TNBC from the AA subpopulation (Supplemental Fig. 1c). Representative images of TOP2A IHC staining are shown in Supplemental Fig. 1d–f. TOP2A was highly expressed on ductal carcinoma in situ (DCIS) and malignant tumor tissue from C3 (1)/Tag mice (Supplemental Fig. 1e, f). However, in normal mammary tissue from wild-type mice, TOP2A was not detected (Supplemental Fig. 1d). These data indicate that the TOP2A gene is overexpressed in TNBCs isolated from both mice and humans.

### Identification of Th1 TOP2A epitopes for vaccine design

**Fig. 1:**
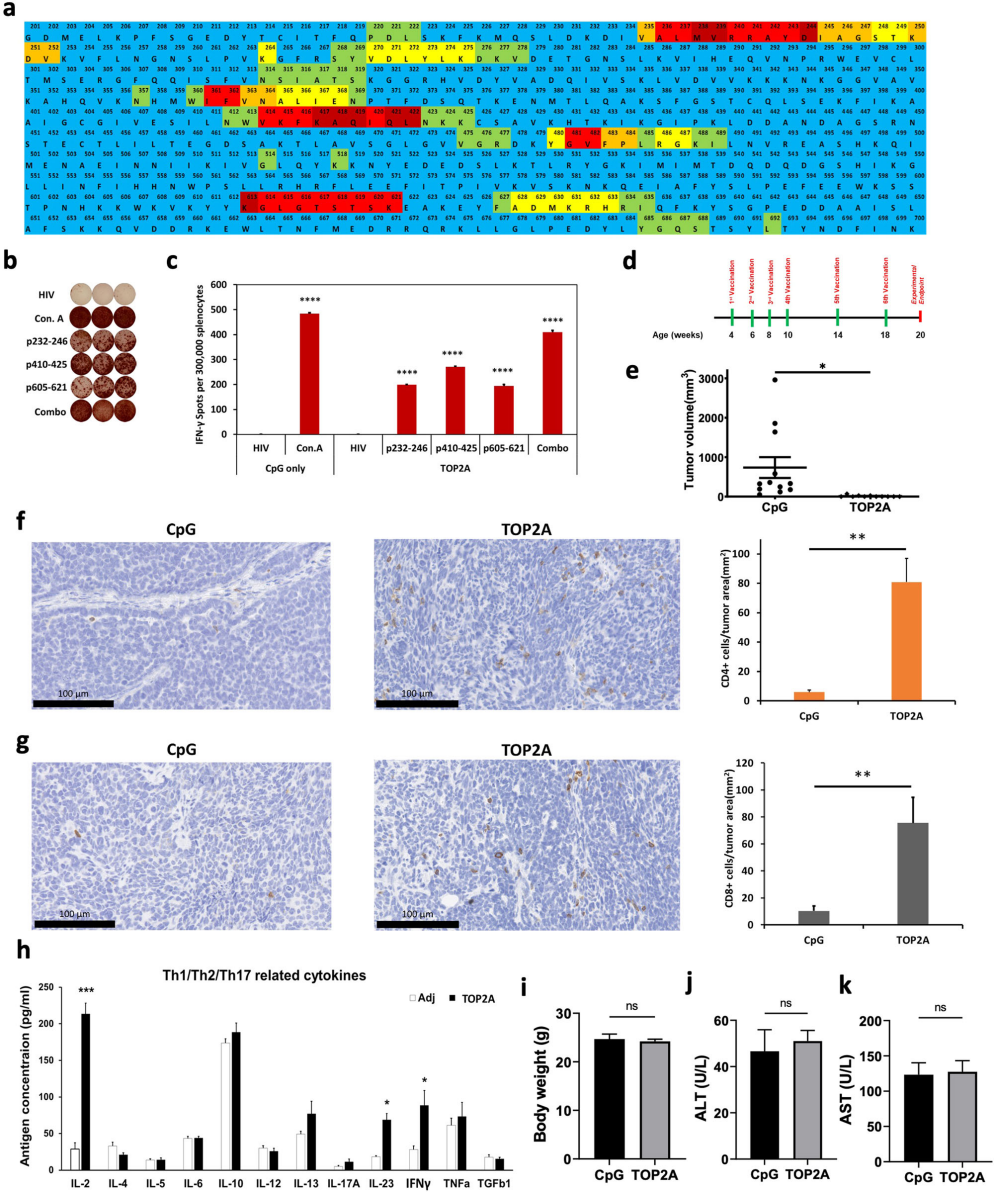
TOP2A vaccination induced the immune response and prevented breast tumor development C3/Tag mice. **a** Representative immunogenic heatmap for TOP2A in FVB mice. The complete mouse TOP2A protein sequence shows identified immunogenic “hot-spots” for FVB mice. Colors represent the percent of the highest score from three algorithms for each amino acid from dark red to light blue in the order of rank scores. **b** Representative IFN-γ based ELISpot assay results showing T cell responses to specific TOP2A peptides from mouse splenocyte. **c** IFN-γ based ELISpot assay results. Splenocytes were collected from vaccinated mice and pulsed with negative control peptide (HIV peptide), positive control peptide (Concanavalin A), 3 individual TOP2A peptides, or their combination (combo). Data are shown as the mean ± SE of three replicate wells per group, *n* = 5, ****p < 0.0001. **d** Experimental design of the experiments. **e** Summary of tumor development in vaccinated C3/Tag genetically engineered mice at 20-weeks of age. Estimated tumor volumes are depicted based on the development of palpable tumors. *p < 0.05. **f** Representative IHC staining and quantitative data for tumor-infiltrationg CD4+ cell from mammary tumor tissues. **g** Representative IHC staining and quantitative data for tumor-infiltrationg CD8+ cell from mammary tumor tissues. The number of positive cells in the field was expressed as # of CD4+ cells (**f**) and CD8+ cells (**g**) per mm^2^ tumor area. **p < 0.01. **h** To evaluate bulk cytokine production by the T cells in culture supernatants. 12 Cytokines were analyzed by an ELISArray Kit, QIAGEN. Splenocytes from either TOP2A vaccinated or adjuvant alone mice were collected and cocultured with TOP2A vaccine for 72 hours, Adj (CpG only, *n* = 3), TOP2A (*n* = 3). **i**–**k** Body weights, ALT and AST level at 20-weeks of age. Data are shown as the mean ± SE, *p < 0.05, ***p < 0.001 (compare with CpG only, 2-tail *t*-test).

Using a multi-scoring system that combines multiple MHC class II peptide binding algorithms, we identified and selected 3 Th1-promoting vaccine candidate peptides, p232 (p232 – p246), p410 (p410 – p425), and p604 (p605 – p621) (Fig. 1a). Immunogenicity of the TOP2A peptides was evaluated in C3(1)/Tag-REAR mice via IFN-γ ELISpot assays (Fig. 1c). Splenocytes from TOP2A vaccinated mice demonstrated strong immune responses with a mean for IFN-γ secreting cells of around 400 spots per well (SPW) compared to less than 10 SPW for negative control HIV peptides. Additionally, we examined the immunogenicity of all three peptides combined (combo). Interestingly, combo-vaccinated mice yielded a significantly stronger immune response than single peptide-vaccinated mice. Among three peptides of TOP2A, two murine peptide sequences (p232-246 and p605-621) exhibited 100% sequence identity with the human TOP2A sequence, while another peptide sequence (p410-425) showed a 93% similarity. These peptides p232, p410, and p604 were chosen to formulate a multi-peptide TOP2A vaccine for the preventive efficacy study.

### TOP2A vaccination prevented TNBC development in a genetically engineered mouse model

To examine tumor preventive effects of the TOP2A vaccine in a more clinically relevant model, we employed the C3(1)/Tag transgenic mouse model which develops invasive mammary gland tumors that share important molecular and biologic features with human basal-like TNBC^17^. The experimental design for these experiments is shown in Fig. 1d. Briefly, mice were vaccinated once every two weeks for four consecutive weeks, and then monthly at 14- and 18- weeks of age. We measured palpable tumor volumes at the end of the experiments (20 weeks). As shown in Fig. 1e, the TOP2A vaccination significantly reduced tumor volume as compared to the adjuvant control (CpG only) with an average tumor size of 736 mm^3^ in adjuvant controls vs. 11 mm^3^ in vaccinated mice (*p* < 0.05). Notably, the vaccine completely prevented mammary gland tumor growth in 8 of the 11 TOP2A vaccinated mice, while all adjuvant controls developed tumors. We confirmed the immunogenicity of TOP2A peptides via the IFN-γ ELISpot assays (Supplemental Fig. 2). We evaluated the number of CD4+ and CD8 + TIL per mm^2^ tumor area in those animals that developed tumors (3 TOP2A vaccinated and 12 CpG control mice) and observed that TOP2A vaccination significantly increased CD4+ and CD8 + TIL over adjuvant controls (Fig. 1f, g; *p* < 0.01). We closely monitored all animals in all groups and body weights, serum ALT and AST levels were not significantly changed in 20 week-age old (Fig. 1i–k). These results demonstrate that TOP2A vaccination can effectively prevent TNBC development in this genetically engineered mouse model and suggest that vaccine-induced tumor antigen-specific T cell responses play a critical role in the immunopreventive effect of the vaccine.

### TOP2A vaccination induced a Th1 cytokine response

To evaluate Th1 and Th2 cytokine production in response to the TOP2A vaccine, we measured the production of 12 cytokines from in vitro peptide-stimulated splenocytes that had been isolated from vaccinated C3(1)/Tag GEM mice (Fig. 1h). The most abundant cytokines detected in response to the TOP2A peptide pool stimulation were Th1 cytokines, IL-2 and IFN-γ, increased approximately 7- and 3-fold, respectively, as compared to adjuvant controls. In contrast, there was no notable increase in Th2 cytokine production (IL-4, IL-5 and IL-13) as compared to the adjuvant controls. Interestingly, the TOP2A vaccine stimulated not only Th1 cytokine production (IL-2 and IFN-γ) but also IL-23 which promotes the differentiation of Th17 lymphocytes. These data suggest that Th1 and Th17 immune responses are predominantly elicited by the TOP2A -specific peptide vaccine.

### TOP2A multi-peptide vaccination slowed the growth of syngeneic mouse TNBC tumors

**Fig. 2:**
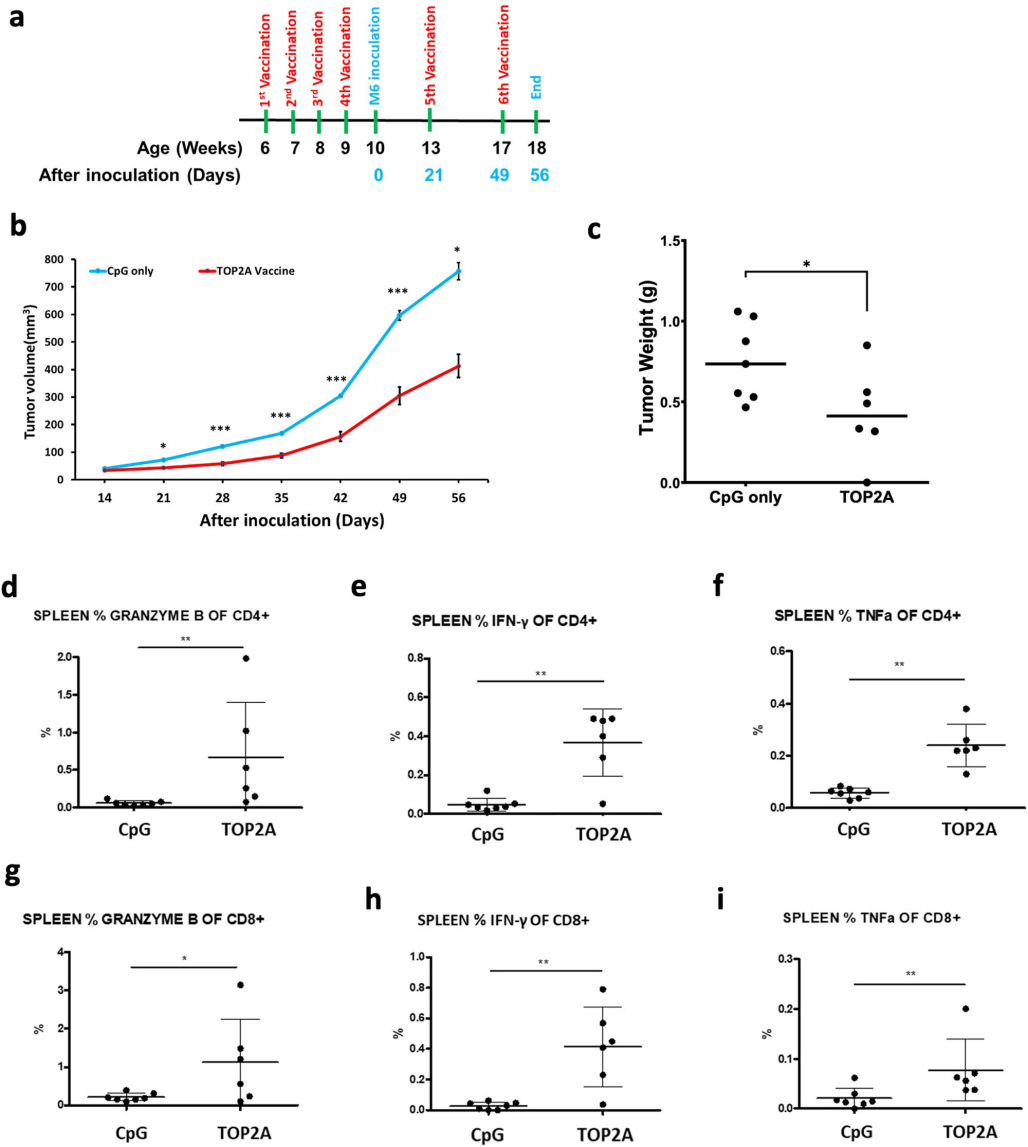
TOP2A vaccination inhibited tumor growth in the syngeneic C3(1)/Tag-REAR mouse model. **a** Experimental design and timeline of vaccine administration. **b** Tumor growth curves after M6 tumor cell inoculation. Following implantation, tumor diameters were measured using a digital caliper and tumor volumes were calculated using the formula: maximum diameter × (minimum diameter)^2^ × 0.4. Data are shown as the mean ± SE, *p < 0.05, **p < 0.01, ***p < 0.001(2-tail *t*-test). **c** The weight of each tumor was taken at the experimental endpoint. Data are shown as the mean ± SE, *n* = 6–7 mice per group, *P < 0.05. **d**–**i** TOP2 A vaccination significantly increased the percentage of the functionally activated CD4+ and CD8+ cells in the spleens. Cells isolated from the spleen were stained for the intracellular markers (granzyme B, IFN-γ and TNFα). *p < 0.05, **p < 0.01 (2-tail *t*-test).

We next examined the antitumor efficacy of TOP2A vaccination in a syngeneic TNBC mouse model, C3(1)/Tag-REAR mice inoculated with M6 tumor cells. As illustrated in Fig. 2a, 7-week old C3(1)/Tag-REAR mice were given an initial vaccination with TOP2A peptides with CpG oligodeoxynucleotide (CpG ODN) adjuvant, followed by three more vaccinations at one-week intervals. One week after the last vaccination, M6 cells were implanted into #4 mammary fat pads of the C3(1)/Tag-REAR mice. Additional vaccinations were administered at four-week intervals until the experimental endpoint. Notably, TOP2A vaccination significantly decreased tumor volumes as compared to CpG-only treated control mice (Fig. 2b; *p* < 0.05). Average tumor size at the experimental endpoint was 757.2 mm^3^ in control vs. 413mm^3^ in vaccinated animals. TOP2A vaccine also reduced tumor weight by nearly 40% (Fig. 2c, *p* < 0.05). TOP2A vaccinated mice were also examined for the presence of a systemic immune response. Splenocytes collected from TOP2A vaccinated mice were stained for the intracellular markers (granzyme B, IFN-γ and TNFα) and analyzed by flow cytometry. Significant increases in CD4 + and CD8 + T cells expressing granzyme B, IFN-γ, and TNFα were observed in the spleens of TOP2A vaccinated animals as compared to controls treated with CpG only (Fig. 2d–i). Taken together, these results indicate that the TOP2A peptide vaccine induced both local and systemic type 1 immune responses.

### TOP2A vaccinated C3(1)/Tag mice were protected from the M6 cell secondary challenge

**Fig. 3:**
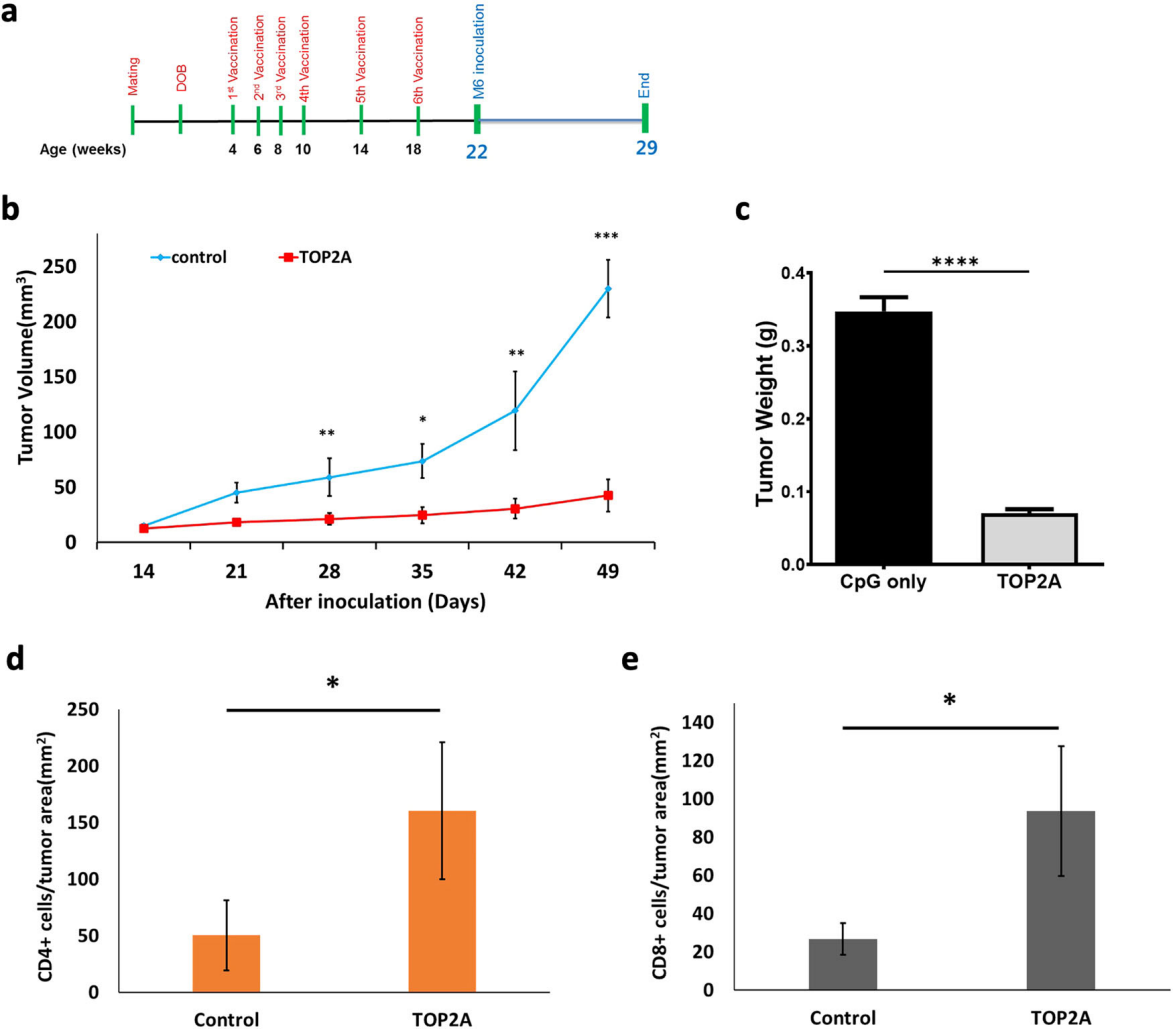
TOP2A vaccinated C3(1)/Tag mice were protected from M6 cell secondary challenge. **a** Schematic of the experimental design and timeline of vaccine administration. **b** Tumor growth curves after M6 tumor cell inoculation in C3(1)/Tag mice at 22 weeks of age. 5 × 10^5^ cells were injected into the #4 mammary fat pads of female C3(1)/Tag mice. Following implantation, tumor diameters were measured using a digital caliper, and tumor volumes were calculated using the formula: maximum diameter × (minimum diameter)^2^ × 0.4. Control C3(1)/Tag female mice (*n* = 4) were never vaccinated. C3(1)/Tag female mice (*n* = 3) were vaccinated with TOP2A. At the time of M6 inoculation, there was no tumor present in the #4 mammary fat pads. **c** Weight of each tumor was taken at the end of the experiment. **d**, **e** TOP2A vaccination increased the number of CD4+ (**d**) and CD8+ (**e**) tumor-infiltrating lymphocytes in treated animals. The number of positive cells in the field was expressed as # of CD4+ and CD8+cells per mm^2^ tumor area. All data are shown as the mean ± SE, *p < 0.05, **p < 0.01, ***p < 0.001(2-tail *t*-test).

We tested the long-term immune memory protective effects of the TOP2A vaccine against secondary tumor cell development. The first “challenge” was the spontaneous tumor that develops in C3/Tag mice (see Fig. 3a for experimental design). Secondary tumor challenge involved transplanting the C3/Tag mice at 22 weeks of age (4 weeks after the final vaccine) with syngeneic M6 tumor cells. Notably, TOP2A vaccinated mice did not develop spontaneous tumors, and the vaccinated mice had significantly smaller M6 TNBC tumors at the experimental endpoint (Fig. 3b, c). Average tumor size in the vaccinated mice as compared to the adjuvant controls was 42 mm^3^ vs. 229.9 mm^3^, respectively (Fig. 3b; *p* < 0.001). In TOP2A vaccinated mice, tumor weight in the TOP2A vaccinated mice was also significantly reduced by nearly 85% (Fig. 3c). The numbers of CD4+ and CD8 + TIL in TOP2A vaccinated mice were also significantly increased, as shown in Fig. 3d. These results suggest that the TOP2A vaccine induces long-term T-cell memory to help resist tumor re-emergence.

### Single cell gene expression landscape in breast tumor and lymph node tissues

We utilized the Seurat package to perform fine clustering of sequenced single cells from mouse breast tumor and lymph node tissues^18,19^. Gene expression data of single cells were aligned and projected in 2-dimensions using uniform manifold approximation and projection (UMAP)^20^. The gene expression patterns of canonical markers were analyzed to characterize different types of immune cell populations in the tumor samples. Six immune cell populations were detected in the breast tumor samples including CD8 + T cells, CD4 + T cells, CD4/CD8 double-negative T cells (DNT), dendritic cells (DC), macrophages and neutrophils (Supplemental Fig. 3a, b). Single cell expression data from the lymph node tissues was also projected by UMAP and immune cell populations identified included CD8 + T cells, CD4 + T cells, DNT, DC and macrophages (Supplemental Fig. 3c, d).

### TOP2A vaccine treatment increased the proportions of tumor specific cytotoxic CD8 + T cells in mouse breast tumor and lymph node tissues

**Fig. 4:**
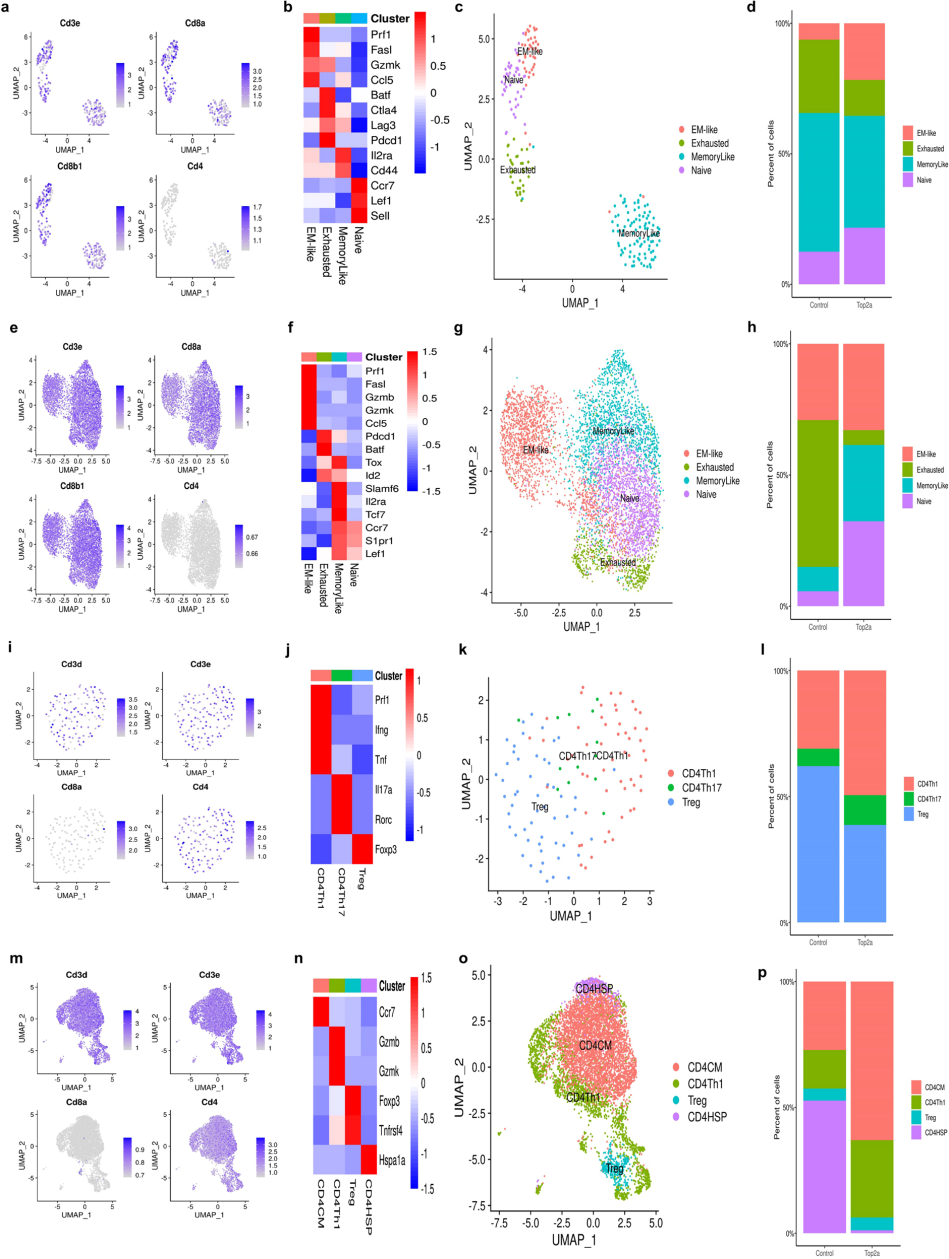
Effects of the TOP2A vaccine on CD8^+^ T and CD4+ T cell subsets in breast tumor and lymph node tissues. **a**, **e** Canonical marker expression singled out CD8^+^ T cell subsets based on scRNA-seq data. **b**, **f** Heatmap of the expression of the markers for each CD8^+^ T cell subset. **c**, **g** The distribution of each CD8^+^ T cell subset; **d**, **h** Percent changes in the CD8^+^ T cell subsets across the control and the TOP2A vaccine treatment groups. **i**, **m** Canonical marker expression singled out CD4 + T cells based on scRNA-seq data. **j**, **n** Heatmap of the expression of the markers for each CD4 + T cell subset. **k**, **o** Distribution of each CD4 + T cell subset; **l**, **p** Percent changes of the CD4 + T cell subsets across the control and the TOP2A vaccine treatment groups.

To determine the effects of TOP2A vaccine treatment on different T cell subsets, we performed deep clustering of CD8 + T cells, CD4 + T cells and DNT cells from mouse breast tumors of 3 of 11 mice that had tumors (8 mice were tumor-free). The location of the CD8 + T cells was identified by the canonical markers (Fig. 4a). Unsupervised clustering of CD8 + T cells using the TILPRED program (https://github.com/carmonalab/TILPRED) identified four CD8 + T cell subsets with distinct transcriptomic profiles (Fig. 4b, c)^21^. The CD8 subsets included effector‐memory (EM)‐like, exhausted, memory-like, and naïve CD8 + T cells. In cancer, the EM-like and exhausted CD8 + T cells are involved in anti-tumor and pro-tumor functions, respectively. TOP2A vaccination greatly increased the abundance of the anti-tumor EM-like CD8 + T cells in the breast tumor microenvironment (TME) (Fig. 4d). In contrast, the exhausted CD8 + T cells were decreased by the TOP2A vaccine treatment. These data suggest that the TOP2A vaccine treatment improves the overall composition of beneficial anti-tumor CD8 + T cells in breast tumors. In the lymph node samples from tumor-bearing mice, we observed similar effects of TOP2A vaccine treatment on CD8 + T cell subsets. CD8 + T cells from the lymph node samples were plotted (Fig. 4e), and CD8 + T cell subsets were identified using corresponding markers (Fig. 4f, g). TOP2A vaccine treatment increased the abundance of EM-like CD8 + T cells and greatly reduced the proportion of exhausted CD8 + T cells in the lymph nodes (Fig. 4h).

### TOP2A vaccine treatment increased the abundance of CD4 + Th1 cells in mouse breast tumor and lymph node tissues

Functional CD4+ cells could play a major role in the anti-tumor response induced by TOP2A vaccine treatment. We analyzed CD4 + T cells from the breast tumors and identified three types of CD4 + T cell subsets including CD4 Th1, CD4 Th17 and Treg cells (Fig. 4i–k). CD4 + Th1 cells overexpressed the marker genes perforin, IFNγ, and TNF; CD4 Th17 T cells overexpressed IL17a and Rorc; Treg cells overexpressed Foxp3 (Fig. 4j). TOP2A vaccine treatment significantly increased the proportion of CD4 + Th1 and Th17 cells in breast tumors while decreasing the abundance of Treg cells (Fig. 4l), suggesting that the anti-tumor function of CD4 + T cells was significantly enhanced by TOP2A vaccine treatment. In the lymph node samples, four types of CD4 + T cells subsets were identified: CD4 CM (central-memory T cells), CD4 Th1, Treg and CD4HSP (CD4 + T cells overexpressing Hspa1a) (Fig. 4m–o). Similar to the breast tumor samples, we observed increased frequencies of CD4 + Th1 cells after TOP2A vaccination (Fig. 4l). CD4 CM cells were also increased, while immune inhibitory CD4 HSP cells were decreased by the TOP2A vaccine treatment (Fig. 4p)^22^. Our results suggest that CD4 + Th1 cells are involved in the anti-tumor response generated by the TOP2A vaccine.

### TOP2A vaccine treatment also impacts other immune cells in mouse tumor and lymph node tissues

We examined tumor and lymph node tissues for changes in other immune cell populations after TOP2A vaccine treatment. For the DNT cells (CD4/CD8 double-negative T cells) in mouse breast tumor and lymph node tissues, the cells were divided into three subsets: helper, cytotoxic, and innate DNT cells (Supplemental Figs. 4, 5)^23^. TOP2A vaccine treatment significantly increased the abundance of cytotoxic DNT cells, while the treatment decreased the proportion of innate DNT cells in mouse breast tumor tissues (Supplemental Fig. 4d). The percentage of helper DNT cells was not altered by TOP2A vaccination (Supplemental Fig. 4d). For the lymph node samples, TOP2A vaccine treatment increased the percentage of helper DNT cells but did not change the percentage of cytotoxic DNT cells (Supplemental Fig. 5d). These results suggest that the TOP2A vaccine treatment specifically increases the abundance of cytotoxic DNT cells in mouse breast tumors. Macrophages were subdivided into M1 and M2 subsets according to their marker gene expression in both breast tumor and lymph node tissues (Supplemental Figs. 6, 7). The abundance of anti-tumor M1 macrophages was increased and the pro-tumor M2 macrophages decreased after TOP2A vaccination in both breast tumor and lymph node tissues (Supplemental Figs. 6d, 7d). These data suggest that macrophages also participate in the anti-tumor response induced by TOP2A vaccination. Two subsets of DC were detected in tissues of treated mice: conventional DC (cDC) and plasmacytoid DC (pDC) based on the analysis of corresponding marker genes (Supplemental Figs. 8, 9)^24^. TOP2A vaccine treatment significantly increased the proportion of cDC in breast tumor tissues but not in lymph nodes. cDC are known to play a critical role in anti-tumor immunity^25^. Finally, we detected neutrophils in the breast tumor samples. Neutrophils can be divided into Stage I and Stage II subsets^26^, which represent progenitor and mature neutrophils, respectively. We identified both neutrophil subsets in breast tumor tissues and found that TOP2A vaccine treatment did not change the proportions of these two subsets (Supplemental Fig. 10a–c).

### TCR clonotype analysis revealed the appearance of new T cell clones as a consequence of TOP2A vaccination

**Fig. 5:**
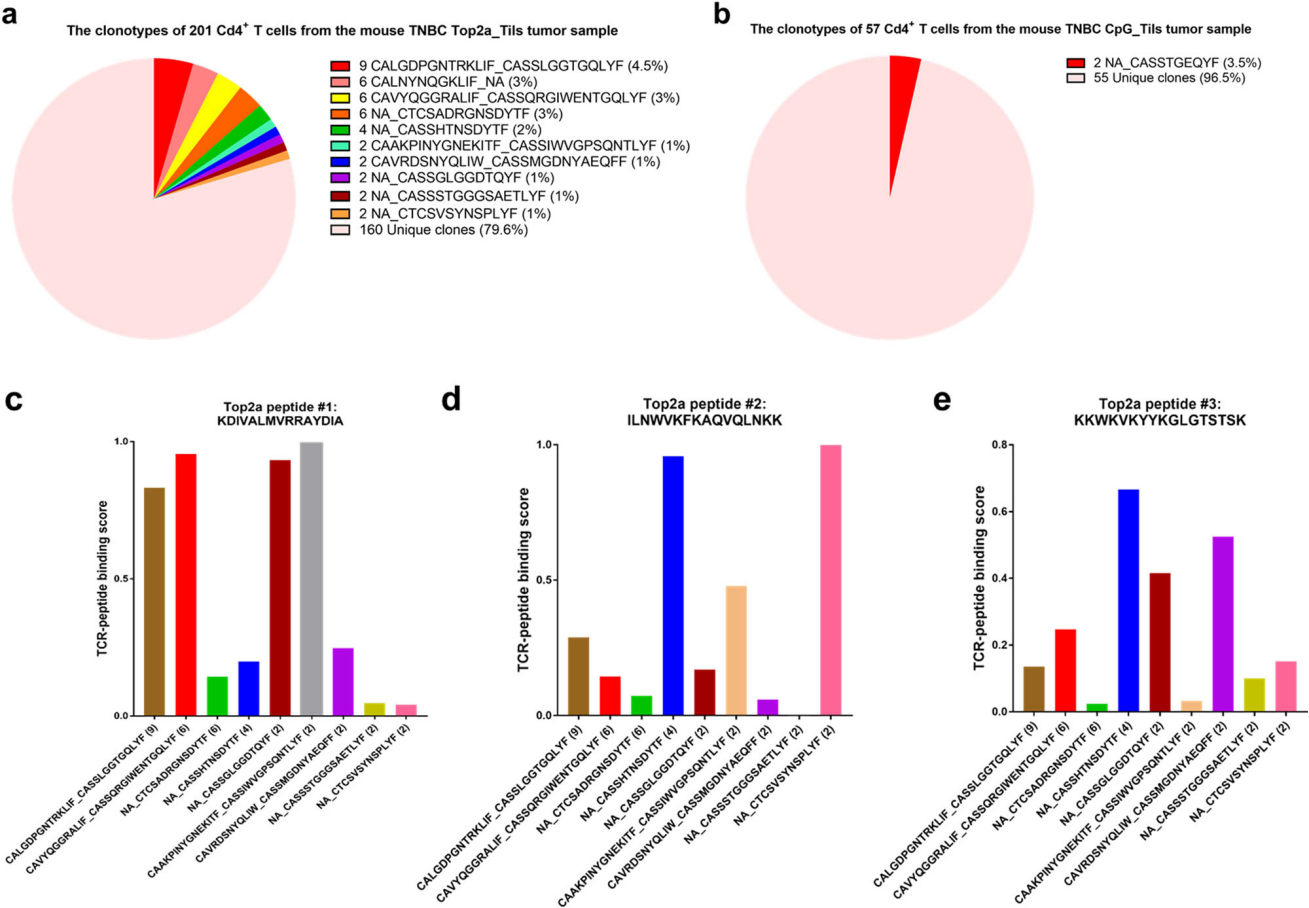
CD4 + TCR clonotype distribution in the mouse breast tumor samples and TCR-peptide binding prediction for the three TOP2A peptide epitopes. **a** Distribution of CD4 + TCR clonotypes in breast tumor tissues from TOP2A vaccine treated mice. **b** Distribution of CD4 + TCR clonotypes in the breast tumor tissues from non-treated CpG control mice. Predicted binding of the following peptides with the CD4 + TCR clonotypes detected in breast tumor tissues from the TOP2A vaccinated mice: **c** First TOP2A peptide – KDIVALMVRRAYDIA; **d** Second TOP2A peptide – ILNWVKFKAQVQLNKK; **e** Third TOP2A peptide – KKWKVKYYKGLGTSTSK.

To explore potential mechanisms underlying the anti-tumor response induced by TOP2A vaccine treatment, we analyzed scTCRseq data of T cells from the control and TOP2A vaccine groups. We sequenced 201 CD4 + T cells in tumor tissues from TOP2A vaccinated mice and 57 CD4 + T cells in tumor tissues from the CpG control mice. The top five most frequent TCR clonotypes (frequency >= 4) constituted 15.5% of the total CD4 + TCR clonotypes in TOP2A vaccine group; these clonotypes were not found in CpG control group, suggesting their involvement in the TOP2A vaccine generated CD4 + T cell immune response (Fig. 5a). Nearly all of the TCR clonotypes in vaccinated mice were unique versus the CpG controls (Fig. 5). To predict the likelihood of TCR-peptide binding for the TOP2A vaccine peptides, we used ERGO (pEptide tcR matchinG predictiOn) software which is a highly specific and generic TCR-peptide binding predictor (https://github.com/louzounlab/ERGO)^27^. For the first TOP2A peptide – KDIVALMVRRAYDIA, the top 4 TCR clonotypes with the highest binding scores include: CAAKPINYGNEKITF_CASSIWVGPSQNTLYF (2 cells, clonotype frequency: 1%), CAVYQGGRALIF_CASSQRGIWENTGQLYF (6 cells, clonotype frequency: 3%), NA_CASSGLGGDTQYF (2 cells, clonotype frequency: 1%), and CALGDPGNTRKLIF_CASSLGGTGQLYF (9 cells, clonotype frequency: 4.5%) (Fig. 5c). For the second TOP2A peptide – ILNWVKFKAQVQLNKK, the top 4 clonotypes with the highest binding scores were: NA_CTCSVSYNSPLYF (2 cells, clonotype frequency: 1%), NA_CASSHTNSDYTF (4 cells, clonotype frequency: 2%), CAAKPINYGNEKITF_CASSIWVGPSQNTLYF (2 cells, clonotype frequency: 1%), and CALGDPGNTRKLIF_CASSLGGTGQLYF (9 cells, clonotype frequency: 4.5%) (Fig. 5d). For the third TOP2A peptide – KKWKVKYYKGLGTSTSK, the top 4 clonotypes with the highest binding scores are: NA_CASSHTNSDYTF (4 cells, clonotype frequency: 2%), CAVRDSNYQLIW_CASSMGDNYAEQFF (2 cells, clonotype frequency: 1%), NA_CASSGLGGDTQYF (2 cells, clonotype frequency: 1%), and CAVYQGGRALIF_CASSQRGIWENTGQLYF (6 cells, clonotype frequency: 3%) (Fig. 5e). Notably, the top two most frequent TCR clonotypes, CALGDPGNTRKLIF_CASSLGGTGQLYF and CAVYQGGRALIF_CASSQRGIWENTGQLYF, both showed high binding affinity to the TOP2A peptide KDIVALMVRRAYDIA (Fig. 5c), suggesting that this TOP2A peptide plays an important role in the TOP2A vaccine-induced anti-tumor T cell response.

## Discussion

In the current study, we developed a multi-peptide vaccine targeting TOP2A and showed that this multi-peptide TOP2A vaccine was strikingly effective in preventive efficacy studies with C3(1)/Tag transgenic mice. Top2A vaccination increased CD4+ and CD8+ tumor infiltrating lymphocytes as well as the percentage of the functionally activated CD4+ and CD8+ cells in the spleens from the vaccinated mice. TOP2A vaccination showed no toxicity when all key organs from the vaccinated C3(1)/Tag mice, suggesting that the TOP2A vaccine is generally safe and does not induce overt autoimmunity. In addition, we showed that a long-term memory response occurs in TOP2A-vaccinated C3(1)/Tag mice by their rejection of a secondary challenge with M6 mammary carcinoma cells.

TOP2A is an enzyme that controls the topological state of DNA. It catalyzes double-stranded DNA breaks and promotes gene transcription during mitosis^28^, and is overexpressed in several malignancies including breast cancer, ovarian cancer and prostate cancer. TOP2A is directly associated with tumor cell proliferation and invasiveness in breast cancer, and expression has been reported to be amplified in TNBC patients with high-risk factors such as large tumor size, high-grade tumor, and lymph node infiltration^14,29,30^. Here, we identified overexpression of the TOP2A gene in both mouse breast tumor cell lines and human TNBC cancer samples (Supplemental Fig. 1). We also confirmed the expression of TOP2A in TNBCs from AA women. The incidence of TNBC has been reported to be higher in AA women and is associated with a poorer prognosis as compared to European American women, presumably due to various sociologic factors in addition to the increased likelihood of developing TNBC^31,32^.

The potential usefulness of any specific animal model of cancer is whether it reasonably parallels the comparable human disease. This is easier to achieve if the cancer is driven by a clearly defined mutation or amplification e.g. pancreatic cancer (KRAS mutation), colon cancers (mutations in APC or WNT pathway), squamous cell skin cancer (p53 mutations), HER2 positive breast cancer (amplification of HER2). In contrast, sporadic TNBC in humans does not have a single driving mutation although the preponderance of tumors has p53 mutations and a loss of RB^15,16^. Tumors derived from the C3(1)/Tag mouse model similarly have loss of function of p53 and RB. Breast Cancer in the C3(1)/Tag mouse model develops through atypical ductal hyperplasia and DCIS starting around 10 weeks of age and progress to mammary adenocarcinomas after 12 weeks of age^33^. Most importantly, tumors from this model appear similar to human TNBC when compared by RNA expression and genomic alterations (amplifications and deletions)^15,16^. Thus, the model appears to be a relatively good model of human TNBC and responds to certain cytotoxic agents which have proven useful against human TNBC. In the present study, TOP2A vaccination was started before cancer development in C3(1)/Tag mice (Fig. 1d), and the vaccine effectively inhibited the tumor volume as compared with the adjuvant control (Fig. 1e). Interestingly tumor incidence was markedly different between the adjuvant control and TOP2A vaccine groups. In TOP2A vaccinated mice, tumors developed in only 27% of mice, whereas all adjuvant-treated controls developed cancer. We observed no TOP2A vaccine-associated toxicities on major organs including the brain, kidney, liver, lung, and bone marrow (data was not shown). Similarly, no toxicities were observed after vaccinating mice with peptide vaccines targeting EGFR, HER2, or IGFBP-2 peptides in humans^9,10,34,35^.

Peptides from any protein expressed within a given cell can be intracellularly processed in the ER and TAP and eventually presented on the cell surface but are normally ignored by the immune system since most of those T cells have been deleted from the T cell repertoire. However, in the case of over-expressed self-proteins, these have been known to drive T cell responses from small percentages of “low” affinity (cryptic) T cell epitopes present in the repertoire. Furthermore, as some of the tumor cells die, the overexpressed proteins can be taken up by APCs and presented to both CD4 & CD8 T cells. CD4 T cells can differentiate into various Th subsets that can induce, modulate and maintain immune responses to tumor antigens. These CD4 T cell subsets include Th1, Th2, and regulatory T cells (Tregs). The Th1 subset produces IFN-γ, TNF-α and IL-2, which regulate cellular immunity and play important roles in anti-tumor immune responses^10^. The TOP2A vaccine employed in the current study induced predominantly Th1 cell responses, without eliciting a strong Th2 response. Type I cytokines secreted by Th1 cells, such as IFN-γ, can up-regulate MHC class I expression on the membrane of tumor cells as well as APC, which can facilitate tumor recognition by CD8 + T cells^36^. Moreover, Th1 cells can also facilitate the cross-presentation of MHC class I binding peptide antigens to CD8 + T cells^10,37^. These mechanisms could be involved in generating the Th1 responses associated with the TOP2A vaccine, and ultimately facilitating the generation of the CD8 + T cell response. It is also possible that Top2A MHC II peptides could also induce immune responses for CD8 + T cells via cross-priming in the vaccinated mice. Furthermore, our MHC Class-II peptides contain two Class-I peptide epitopes: P235(VALMVRRAY) within Class-II peptide P232 and P414 (VKFKAQVQL) within Class-II peptide P410. Cytotoxic CD8 + T cells play an important role in the anti-tumor immune response by direct killing of tumor cells^38^. Our study demonstrated that the TOP2A vaccine could stimulate both local and systemic antigen specific CD4+ and CD8 + T cell responses. We also found a significant increase in IL-23 secretion from splenocytes isolated from TOP2A vaccinated mice. IL-23 is important and necessary for the differentiation of Th17 lymphocytes^39^. This is notable as IL-23 is known to play a role in antitumor activity^40,41^.

In summary, we showed here that a TOP2A multi-peptide-based vaccine has preventive activity in a TNBC mouse model. TOP2A vaccination induced a Th1 immune response that significantly decreased TNBC tumor formation in both syngeneic and GEM models. Meanwhile, no toxicity was observed in vaccinated mice. We designed 15–17 amino acid peptides with 100% sequence identity between human and mouse TOP2A, making it possible that these peptides could be directly translated into clinical studies. Overall, our data demonstrate that the multi-peptide TOP2A vaccine is highly immunogenic and has the potential to be efficacious in the immunoprevention of TNBC in humans.

## Methods

### Mice

Transgenic C3 (1)/Tag mice and C3(1)/Tag-REAR (abbreviation for rearrangement) mice were a generous gift from Dr. Jeffery E. Green^17^. FVB/N wild-type mice were bought from the Jackson Laboratory. For all experiments, only F2 C3(1)/Tag or C3(1)/Tag-REAR generation mice were used. Mice were acclimatized one week following arrival to the facility. All mice were euthanized through CO_2_ inhalation in the mouse cage and followed by cervical dislocation when experiments reached at end point. Mice were maintained and bred in the Biomedical Resource Center at the Medical College of Wisconsin (MCW), Milwaukee, WI and the Houston Methodist Research Institute, Houston, TX. All procedures were approved by the Institutional Animal Care and Use Committee (IACUC).

### Cell lines

M27 (weakly tumorigenic/benign tumor), M6 (malignant tumor), and M6C (metastatic tumor) and M28 (normal control) cells, all derived from C3 (1)/Tag mice, were provided by Dr. Jeffery E. Green^42^. The cell lines were maintained in DMEM medium containing high glucose (Gibco), supplemented with 5% fetal bovine serum, penicillin/ streptomycin and sodium pyruvate (Invitrogen).

### Immunohistochemistry (IHC)

IHC staining was performed by the Children’s Research Institute Histology Core at MCW. Leica Bond Immunostainer Max (model # 10897664) and Leica Bond Immunostainer RX system (model #11784892) were used for human tissue microarray (TMA) and murine samples, respectively. Mouse mammary gland samples from C3(1)/Tag or C3(1)/Tag-REAR mice were formalin-fixed and paraffin-embedded (Sakura Tissue Tek VIP5). Four 4μm sections were used for IHC staining. The numbers of CD4+ and CD8+ tumor-infiltrating T-lymphocytes (TIL) were determined as cells per mm^2^ tumor area by using CD4 antibody (Invitrogen 14-9766-82) and CD8 antibody (Invitrogen 14-0808-82).

### ELISpot assay

Cell suspensions from whole spleens were filtered through a 70 μm cell strainer (BD) and subjected to red blood cell lysis using ACK lysis buffer. 3.0 × 10^5^ cells were plated into individual wells of a MAIPS4510 multiscreen 96-well plate coated with anti-interferon γ (IFN-γ) detection antibody and containing media with either peptide, concanavalin A (positive control), HIV peptide (negative control), or no antigen (negative control). After 72-hour incubation, plates were washed and incubated with a secondary antibody (BD) overnight at 4 °C. Wells were then washed with PBS and HRP streptavidin was added. Following one-hour incubation, the plate was developed using AEC substrate for between five to 25 minutes. An automated plate reader system (CTL Technologies) was used to image the plates and quantify spot numbers.

### Scoring system for the prediction of MHC class II binding epitopes

A combined scoring system published by Dr. Disis and colleagues was used to identify selected antigen-specific MHC epitopes with optimal binding affinity^34^. Briefly, to identify antigen-specific MHC class II epitopes with optimal binding affinity and promiscuity across multiple alleles, the following algorithms were used for prediction: NetMHCIIpan (https://services.healthtech.dtu.dk/service.php?NetMHCIIpan-4.0, Technical University of Denmark, Lyngby, Denmark) and Rankpep (http://imed.med.ucm.es/Tools/rankpep.html, University Computense Madrid, Harvard, Madrid, Spain). For each available MHC class II allele, 20 peptide sequences were initially selected solely based on the rank-order of the predicted binding affinity from each algorithm. The sequences are approximately 15 amino acids in length. Individual amino acids for each selected peptide were assigned a score, with one being an amino acid contained in a peptide sequence that ranked highest for predictive binding affinity. Scoring individual amino acids accounted for the multiple-peptides overlaps occurring within and across algorithms. The scores (S) for each amino acid were summed up across the multiple MHC class II alleles from two algorithms. Then, the number (N) of MHC class II alleles, for which each amino acid was predicted to have high-affinity binding, was counted. The final score for each amino acid was calculated by multiplying S and N. For ease of identifying the most potentially immunogenic segments of the protein, each amino acid was assigned a color (from dark red to light blue) based on its final score percentile, with dark red being highest at ≥75% and light blue the lowest at <10%. The color strata are as follows: dark red ≥75% of highest score; red = 50~75% of highest score; orange = 40~50% of highest score; yellow = 30~40% of highest score; green = 20~30% of highest score; blue ≤20% of highest score. Thus, the dark red color corresponds to a sequence where multiple peptides scored highly within an algorithm as well as across algorithms. Light blue represents sequences that are the least potentially immunogenic of all predicted high-binding peptides.

### Vaccine preparation and immunization

A total of 150 μg of vaccine, including 50 μg of each individual peptide, was administered to the mice. Three different TOP2A peptides were purchased from Genemed Synthesis and diluted in phosphate-buffered saline (PBS) to 50 μl/mouse. Peptides and equal amounts of adjuvant CpG (Class B CpG oligonucleotide; a murine TLR9 ligand, Cat. No. tlrl-1826, InvivoGen) were added to bring the total vaccine volume to 100 μl/mouse. CpG was used at 50 μg per mouse. Mice were injected subcutaneously with TOP2A vaccines following the timelines shown in Figs. 1d, 2a, 3a.

### In vivo tumorigenicity assay

In the syngeneic model, C3(1)/Tag-REAR mice were generated from a C3(1)/Tag founder line through the loss of one of the original multiple copies of the C3(1)/Tag-antigen transgene resulting in no spontaneous cancer phenotype^17^. M6 cells, derived from a C3(1)/Tag transgenic mammary tumor, were implanted into the mammary fat pad of C3(1)/Tag-REAR mice. M6 cells were washed, resuspended in PBS at a density of 1 × 10^6^ cells in 100 μl PBS, and injected into the #4 mammary fat pads of female C3(1)/Tag-REAR mice. Following implantation, tumor diameters were measured using calipers and tumor volumes were calculated using the formula: maximum diameter × (minimum diameter)^2^ × 0.4. In the spontaneous GEM model, C3(1)/Tag mice were treated with the TOP2A peptide vaccine following the experimental design in Fig. 1d. C3(1)/Tag mice were euthanized at 20-weeks of age for estimation of tumor development. Tumor volumes were measured using calipers and calculated using the formula: maximum diameter × (minimum diameter)^2^ × 0.4.

### Cytokine analysis

Mouse Th1/Th2/Th17 Cytokines Multi-AnalyteELISArray™ Kits (Qiagen) were used for cytokine analysis. The cytokines represented by this array are IL2, IL4, IL5, IL6, IL10, IL12, IL13, IL17A, IL23, IFN-γ, TNFα, and TGFβ1. Splenocytes from different groups of mice were stimulated with different peptides for 72 hours, and then culture supernatants were collected and assayed based on the manufacturer’s instructions.

### Flow cytometry

Cell pellets were incubated with surface markers of interest at the recommended or titrated concentrations, incubated at 4 °C for 30 minutes, and protected from light. After incubation, cells were washed and resuspended in FACS fixation buffer for either analysis or intracellular staining. To begin intracellular staining, cells were fixed with Foxp3/Transcription factor staining buffer set (eBioscience) and stained with intracellular markers of interest at the recommended or titrated concentrations at 4 °C for at least 30 minutes while protecting them from light. Samples were washed with permeabilization buffer and resuspended in FACS fixation buffer. Stained cells were fixed in 1% paraformaldehyde and permeabilized following the manufacturer’s instructions to evaluate the expression of intracellular targets, granzyme B, IFN-γ and TNF-α. Flow cytometry was conducted using an LSR-II flow cytometer (BD). Data were analyzed using FlowJo software (Tree Star). FACS sequential gating/sorting strategies are provided in Supplementary Fig. 11.

### Single-cell RNA sequencing (scRNAseq) and TCR sequencing (scTCRseq)

For scRNAseq, randomly selected lymph node samples (1 sample from CpG control group and 2 samples from TOP2A vaccine treated group) and the breast tumor samples (1 sample from CpG control group and 1 sample from TOP2A vaccine treated group) of C3(1)/Tag mice were harvested at the end of the study, minced and digested at 37 °C for 30 minutes with mouse tumor dissociation buffer (Miltenyi Biotec, CA) to generate single-cell suspensions per the manufacturer’s instructions. The processed samples were directly stained with violet viability dye, APC anti-CD45, and CD45+ leukocytes were sorted using FACS. FACS-sorted CD45+ leukocytes were then spun down at 300 *g* for 5 minutes and counted manually with a Neubauer Chamber. Approximately 2.0 × 10^4^ cells were loaded onto the 10X Chromium Controller per the manufacturer’s instruction, resulting in a recovery of about 1 × 10^4^ cells. For the lymph node samples, the libraries of single-cell transcriptome were generated by Chromium Single Cell 3’ v3 Reagent Kits (10x Genomics). For tumor samples, single-cell transcriptome and single-cell TCR libraries were prepared using a 10x Chromium Single-cell 5′ and VDJ library construction kit. All of the libraries were sequenced using NextSeq 500/550 High Output Kits v2 (150 cycles) (Illumina) according to the manufacturer’s protocol.

### scRNA-seq data analysis

Raw sequencing data were de-multiplexed and converted to gene-barcode matrices using the Cell Ranger (version 2.2.0) mkfastq and count functions, respectively (10x Genomics). The mouse reference genome mm10 was used for alignment. Data were further analyzed in R (version 3.4.0) using Seurat (version 3). The number of genes detected per cell, number of unique molecular identifiers (UMIs), and the percent mitochondrial genes were plotted, and outliers were removed to filter out doublets and dead cells. Raw UMI counts were normalized and log transformed. Integrated analysis was then performed to identify shared cell clusters that are present across different datasets. Principal component analysis was performed using variable genes, and the top 20 most statistically significant principal components were used for UMAP analysis.

### Statistical analysis

All in vitro assays were performed at least in triplicate. Six to twelve mice per group were used for the in vivo studies. A two-tailed Student’s *t*-test was used to evaluate differences between the control group and each treatment group. *P*-values < 0.05 were considered statistically significant.

### Reporting summary

Further information on research design is available in the Nature Research Reporting Summary linked to this article.

### Supplementary information

Supplemental Figures

Source data for experiments

REPORTING SUMMARY

## Data Availability

The data that support the findings of this study are available from the corresponding author upon reasonable request. The source data underlying Figs. 1e–k, 2b–I and 3b–d and Supplemental Fig. 2b are provided as a Source Data file. The raw sequence data generated in this paper have been deposited into the NCBI Sequence Read Archive (SRA) under BioProject accession number PRJNA1001015.
