# Supplementary material for: Striking efficacy of a vaccine targeting TOP2A for triple-negative breast cancer immunoprevention
Source: NPJ Precis Oncol. 2023 Oct 25;7:108. doi: 10.1038/s41698-023-00461-1 (PMC10600249; doi:10.1038/s41698-023-00461-1)
Supplement: Supplementary file 1 — Supplemental Figures [file 41698_2023_461_MOESM1_ESM.pdf]

Supplemental Figure. 1

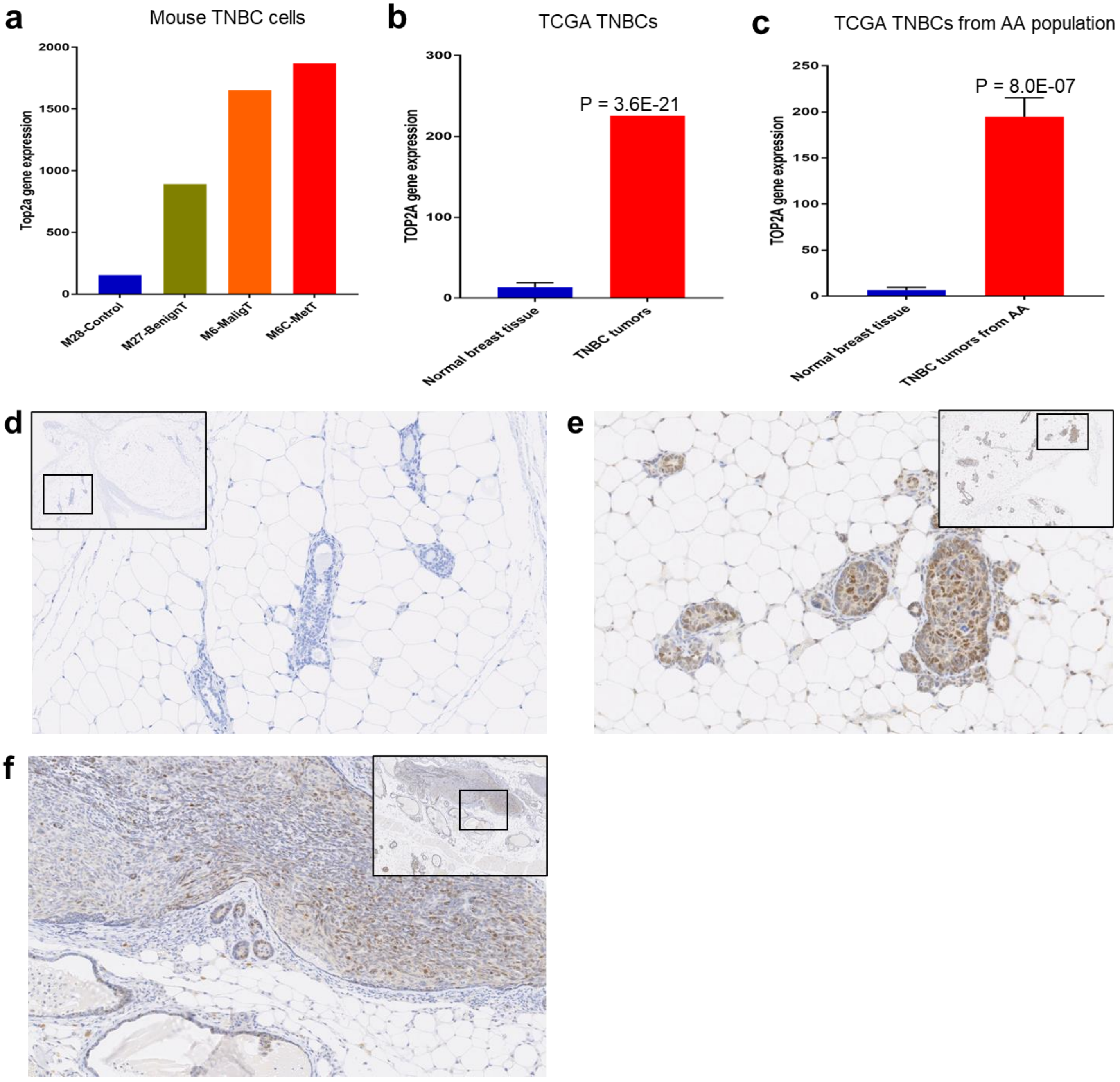

Supplemental Fig. 1 Overexpression of the TOP2A gene in mouse and human TNBC tissues. a Overexpression of the TOP2A gene in M6 mouse mammary carcinoma cells vs. M28 normal mammary cells; both cell lines were derived from C3(1)/Tag mice. **b** Overexpression of the TOP2A gene in human TNBCs. **c** Overexpression of the TOP2A gene in African American (AA) TNBCs. d - f Immunohistochemistry of TOP2A expression on the mammary gland tissue of C3(1)/Tag mice. (d) Expression in mammary gland tissue from a wild-type littermate of the C3(1)/Tag mice. (e) Expression in ductal carcinoma in situ (DCIS). (f) Expression in invasive carcinoma. All images are scanned and captured by NanoZoomer system (Hamamatsu Photonics, Hamamatsu, Japan)

Supplemental Fig. 2

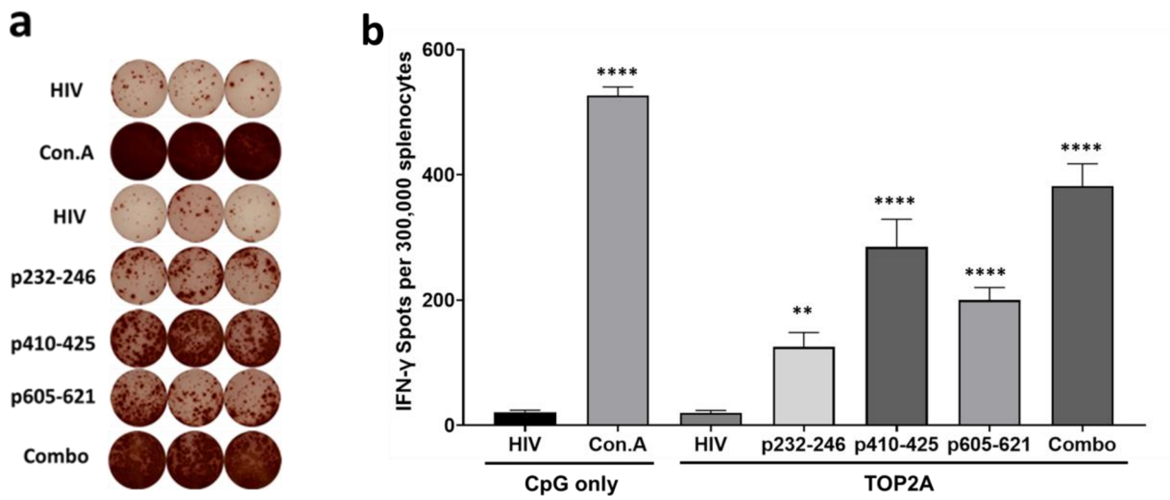

Supplemental Fig. 2 IFN-γ based ELISpot assay on C3(1)/Tag mice. **a** Representative IFN-γ based ELISpot assay results showing T cell responses to specific TOP2A peptides from mouse splenocytes. **b** IFN-γ based ELISpot assay results. Splenocytes were collected from vaccinated mice and pulsed with negative control peptide (HIV peptide), positive control peptide (Concanavalin A), 3 individual TOP2A peptides, or their combination (combo). After 72h of incubation, the ELISpot assay was performed, plates were scanned, and spot numbers were statistically analyzed. Data are shown as the mean ± SE of three replicate wells per group, n=5, **, *p*<0.01****, *p*<0.0001 (2-tail t-test).

Supplemental Fig. 3

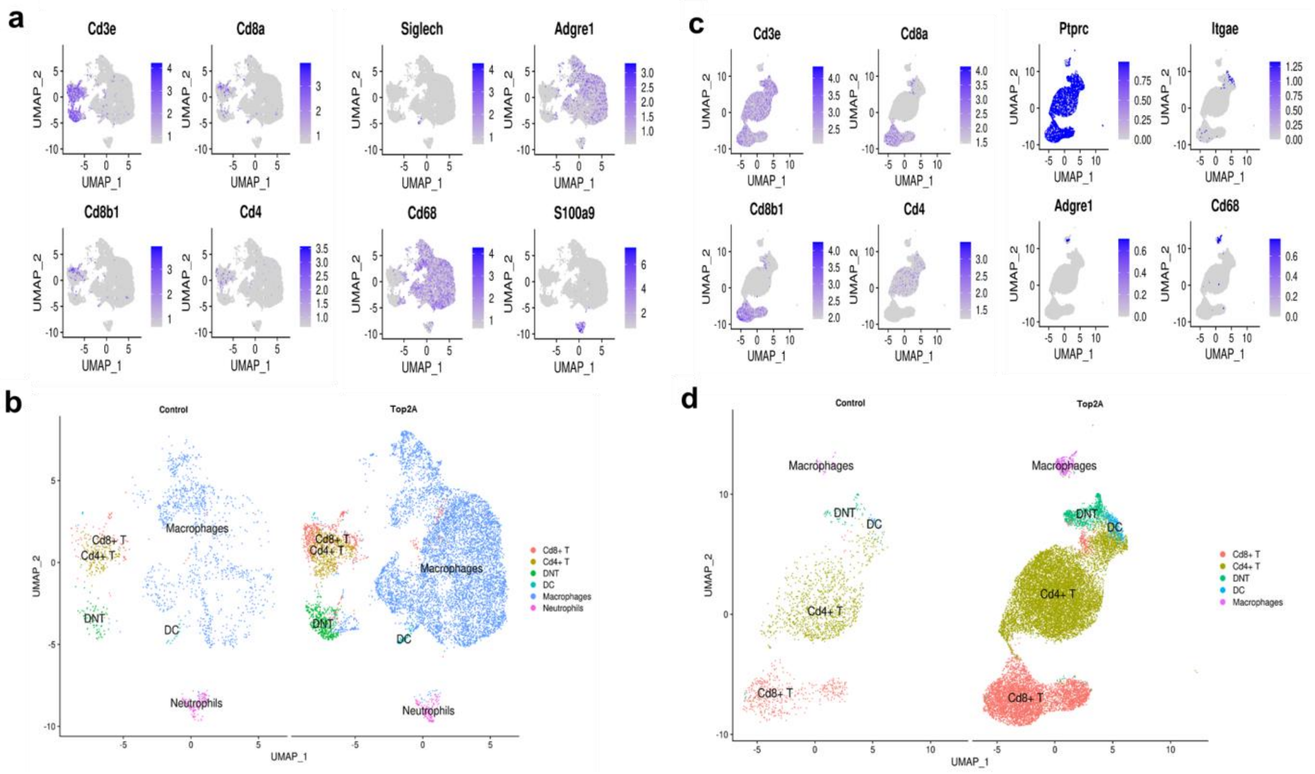

Supplemental Fig. 3 Clustering analysis of scRNA-seq data from TNBC and lymph node tissue immune cells sorted by flow cytometry. **a** The expression of the marker genes for the CD8+ T, CD4+ T, DNT, DC, macrophages and neutrophils in mouse TNBC tumor samples from control TOP2A vaccinated mice. **b** Landscape of the overall immune cell populations from the tumor samples of control and TOP2A vaccinated mice. **c** Expression of the marker genes for the CD8+ T, CD4+ T, DNT, DC and macrophages in the lymph node samples from control and TOP2A vaccinated mice. **d** Landscape of the immune cells in lymph nodes of control and TOP2A vaccinated mice.

Supplemental Fig. 4

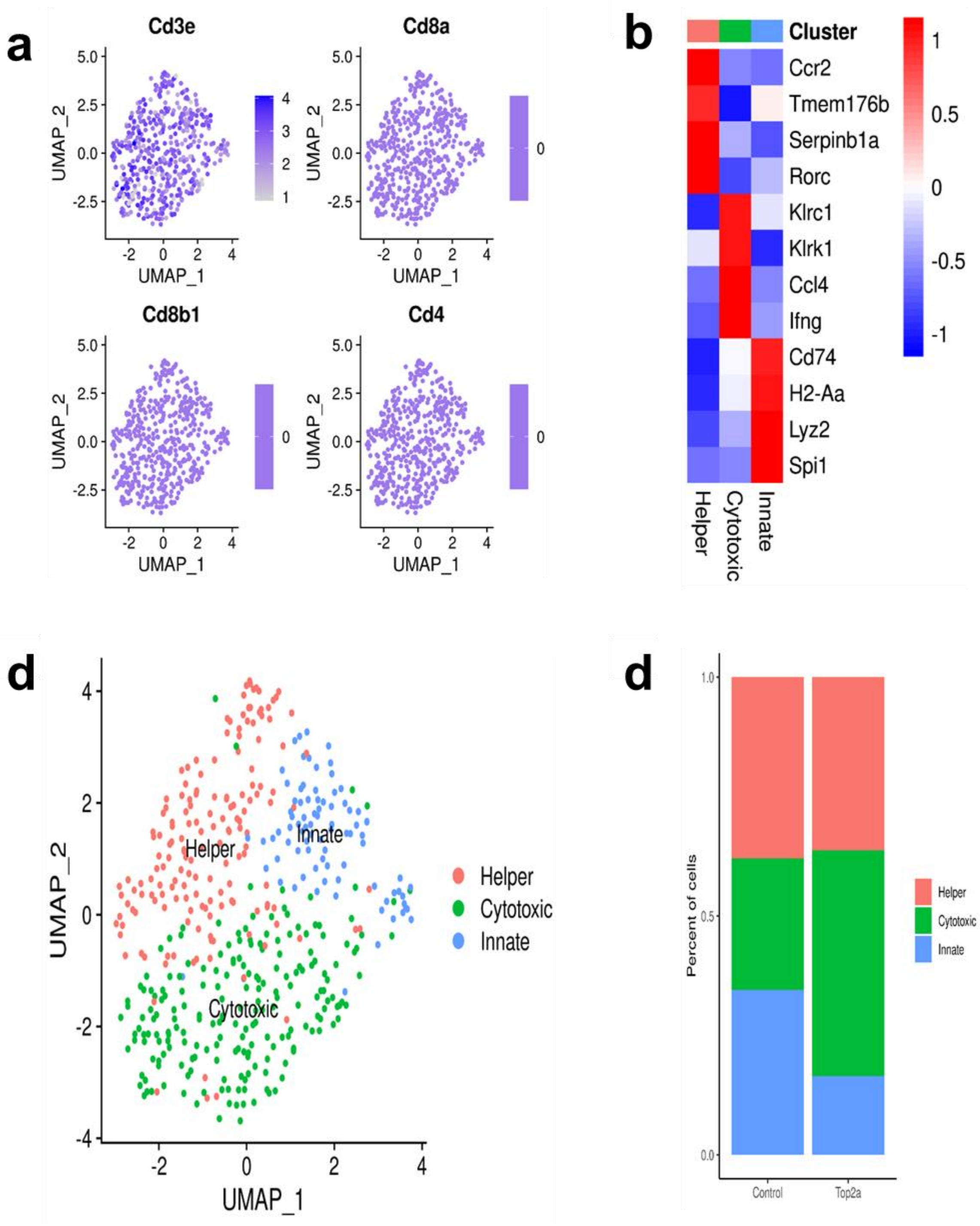

Supplemental Fig. 4 Effects of TOP2A vaccine treatment on the DNT cells (CD4-CD8- double negative T cells) in breast tumor samples. a Canonical T cell marker expression was used to generate UMAP plots from scRNA-seq data. **b** Heatmap of the expression of the markers for each DNT cell subset. **c** Distribution of each DNT cell subset. **d** Percent changes of the DNT cell subsets across the control and the TOP2A vaccine treatment groups.

Supplemental Fig. 5

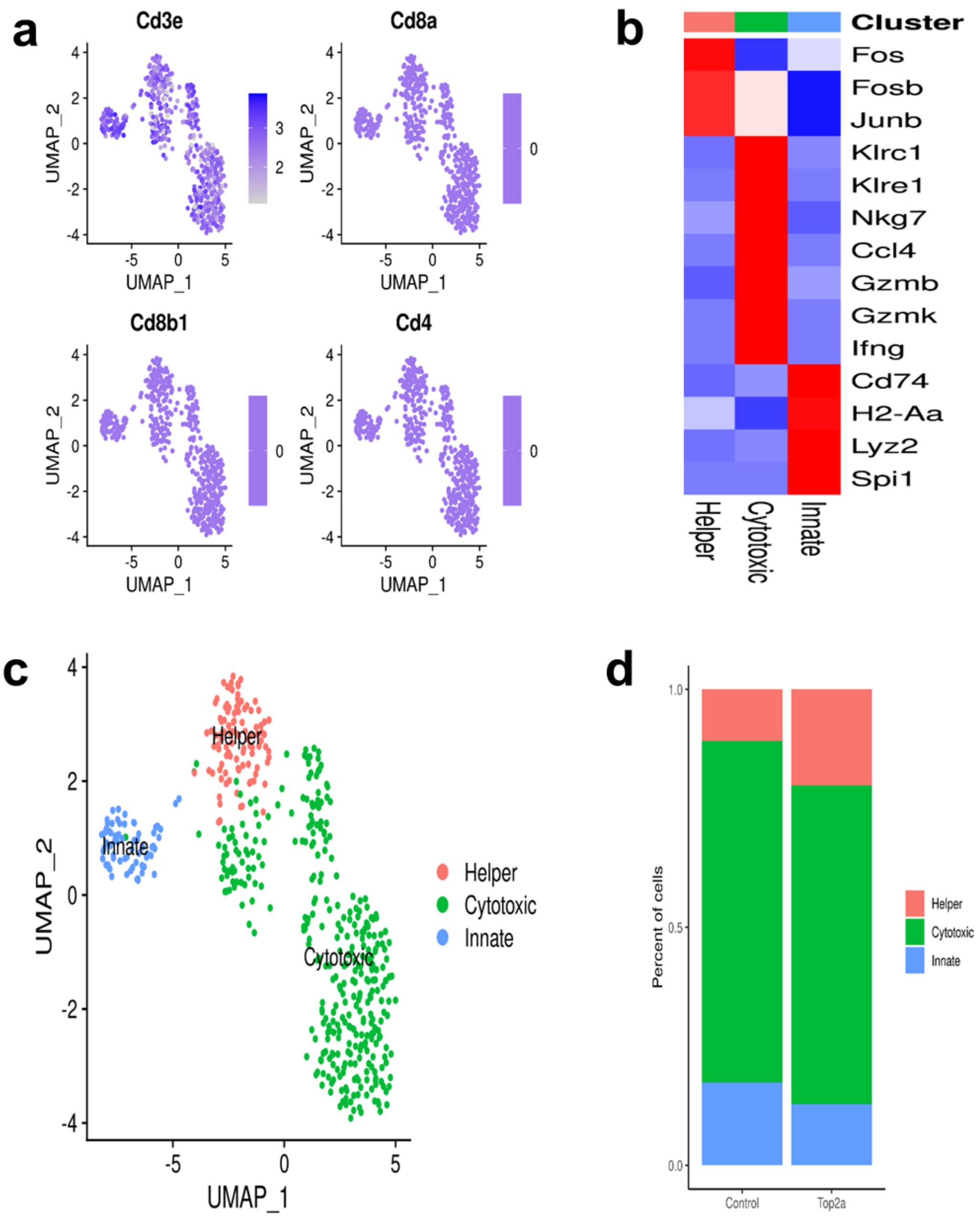

Supplemental Fig. 5 Effects of TOP2A vaccine treatment on DNT cell subsets in lymph node tissues.
a Canonical marker expression on DNT cells was used to generate UMAP plots from scRNA-seq data. **b** Heatmap of markers expressed by each DNT cell subset. **c** UMAP plot distribution of each DNT cell subset. **d** Percent changes in the DNT cell subsets across the control and the TOP2A vaccine treatment groups.

Supplemental Fig. 6

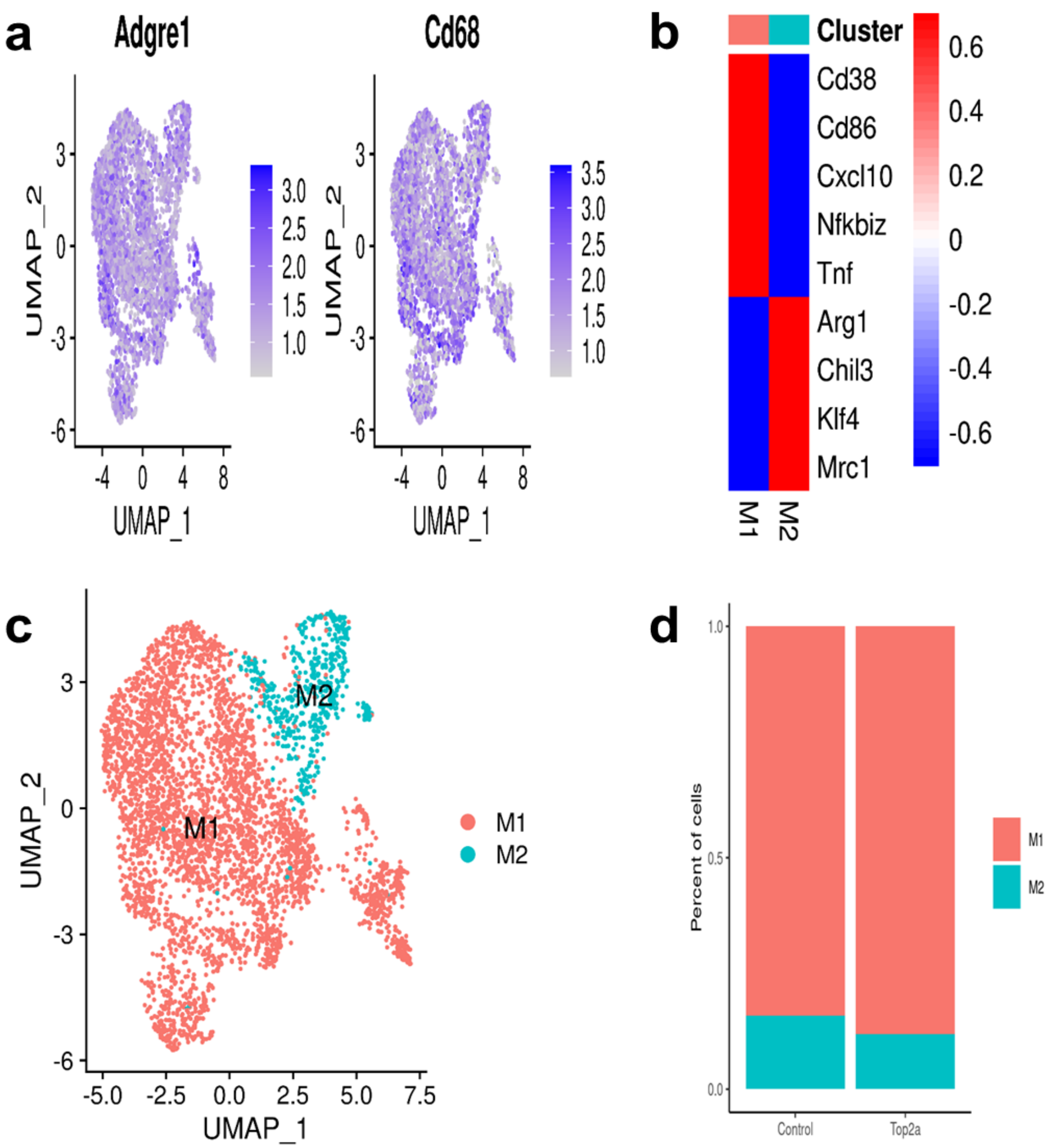

Supplemental Fig. 6 Effects of the TOP2A vaccine on macrophages in the breast tumor samples. a Canonical markers expressed on macrophages were used to generate UMAP plots based on scRNA-seq data. **b** Heatmap based on the expression of the markers for each macrophage subset, M1 (anti-tumor) and M2 (pro-tumor) macrophages. **c** UMAP plot distribution of the M1 and M2 macrophage subsets. **d** Percent changes in the M1 and M2 macrophage subsets in control versus TOP2A vaccine-treated mice.

Supplemental Fig. 7

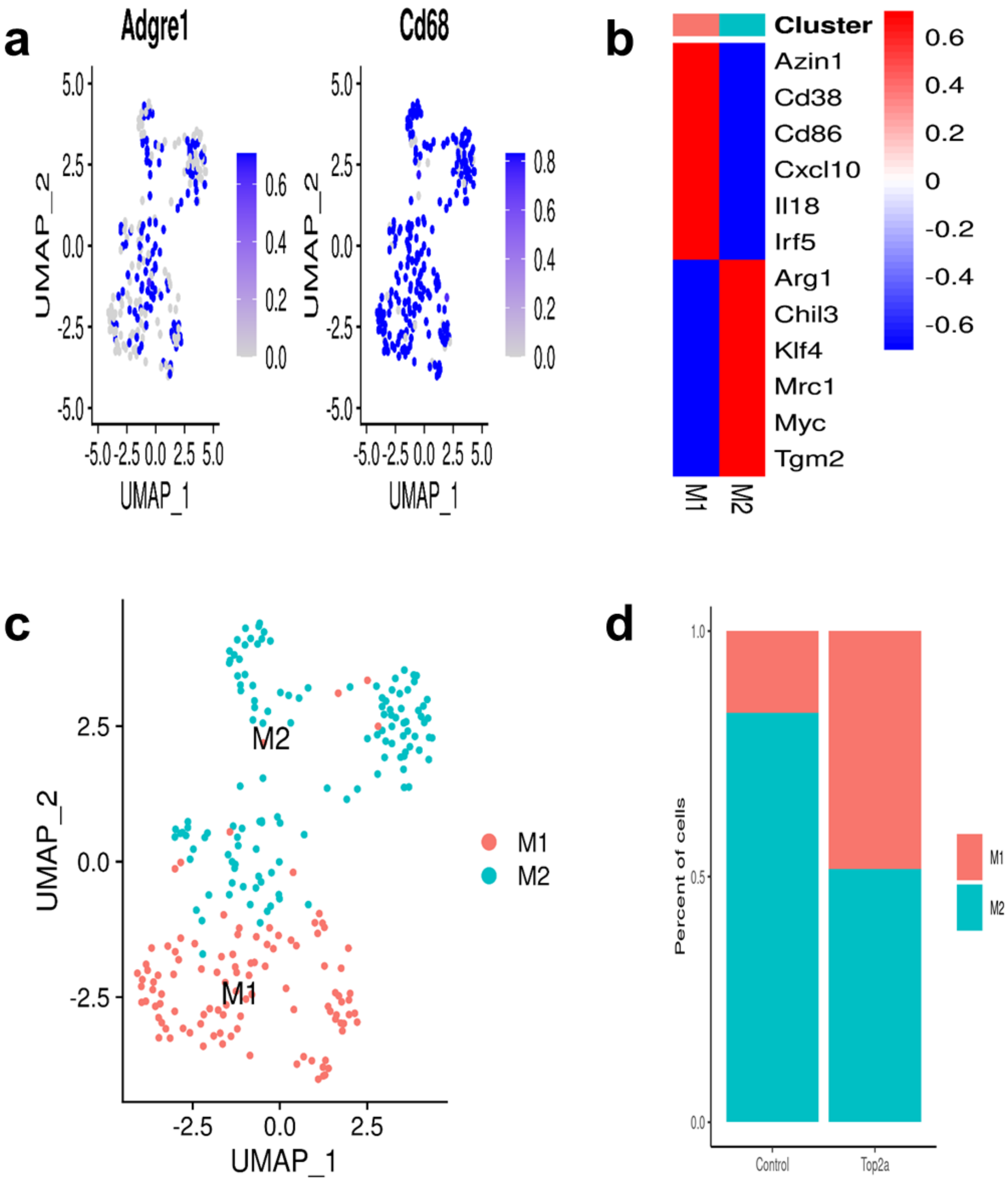

Supplemental Fig. 7 Effects of TOP2A vaccine treatment on macrophages in lymph nodes. a Canonical markers expressed on macrophages were used to generate UMAP plots based on scRNA-seq data. **b** Heatmap based on the expression of the markers for M1 and M2 macrophage subsets. **c** UMAP plot distribution of the M1 and M2 macrophage subsets. **d** Percent changes in the M1 and M2 macrophage subsets in control versus TOP2A vaccine-treated mice.

Supplemental Fig. 8

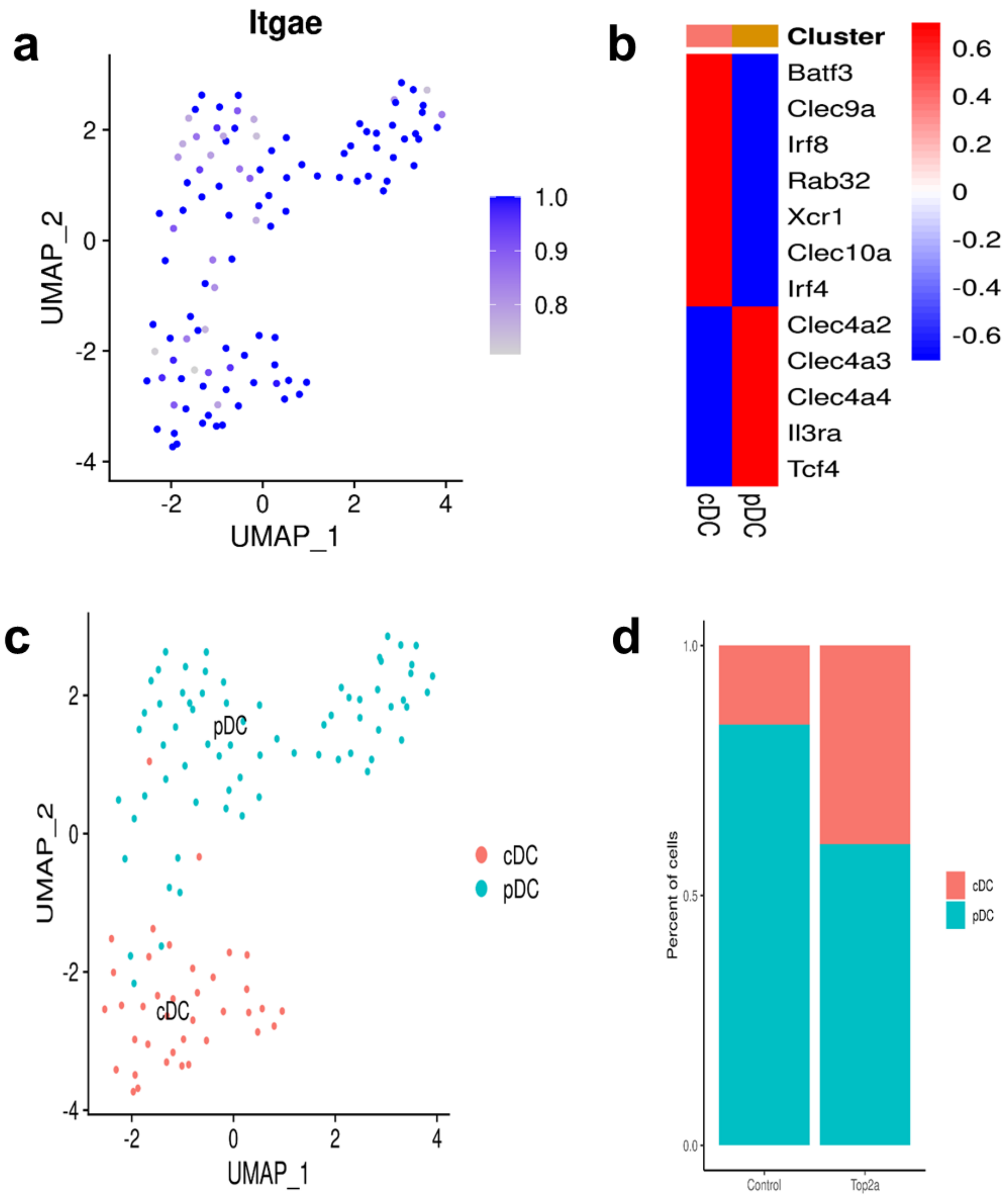

Supplemental Fig. 8 Effects of the TOP2A vaccine on dendritic cells (DC) in the breast tumor samples. a Canonical markers expressed on DC were used to generate UMAP plots based on scRNA-seq data. **b** Heatmap based on the expression of the markers for each DC subset, conventional dendritic cells (cDC) and plasmacytoid dendritic cells (pDC). **c** UMAP plot distribution of the cDC and pDC subsets. **d** Percent changes in the cDC and pDC subsets in control versus TOP2A vaccine-treated mice.

Supplemental Fig. 9

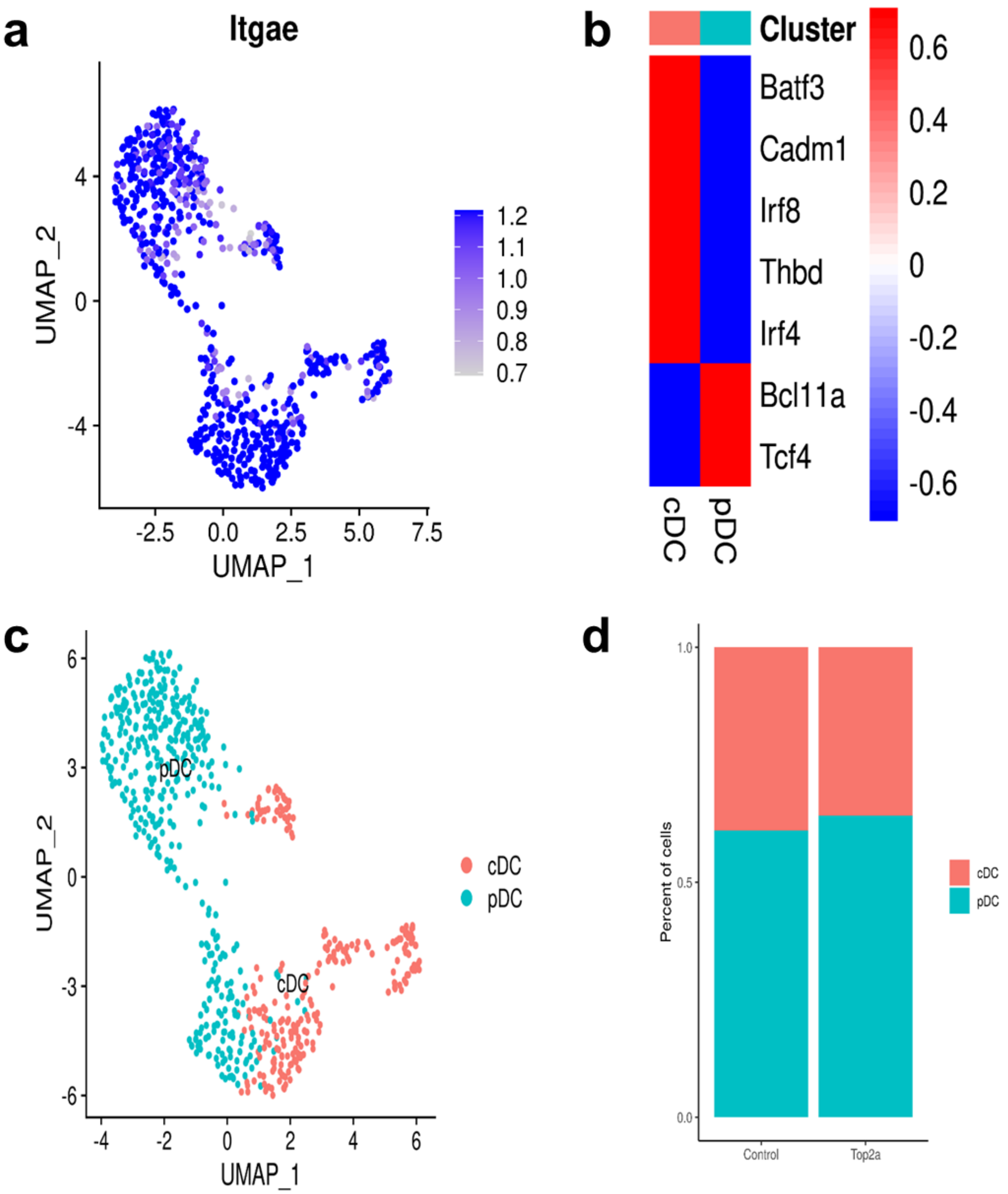

Supplemental Fig. 9 Effects of TOP2A vaccine treatment on DC in the lymph node samples. a Canonical markers expressed on DC were used to generate UMAP plots based on scRNA-seq data. **b** Heatmap based on the expression of the markers for cDC and pDC subsets. **c** UMAP plot distribution of the cDC and pDC subsets. **d** Percent changes in the cDC and pDC subsets in control versus TOP2A vaccine-treated mice.

Supplemental Fig. 10

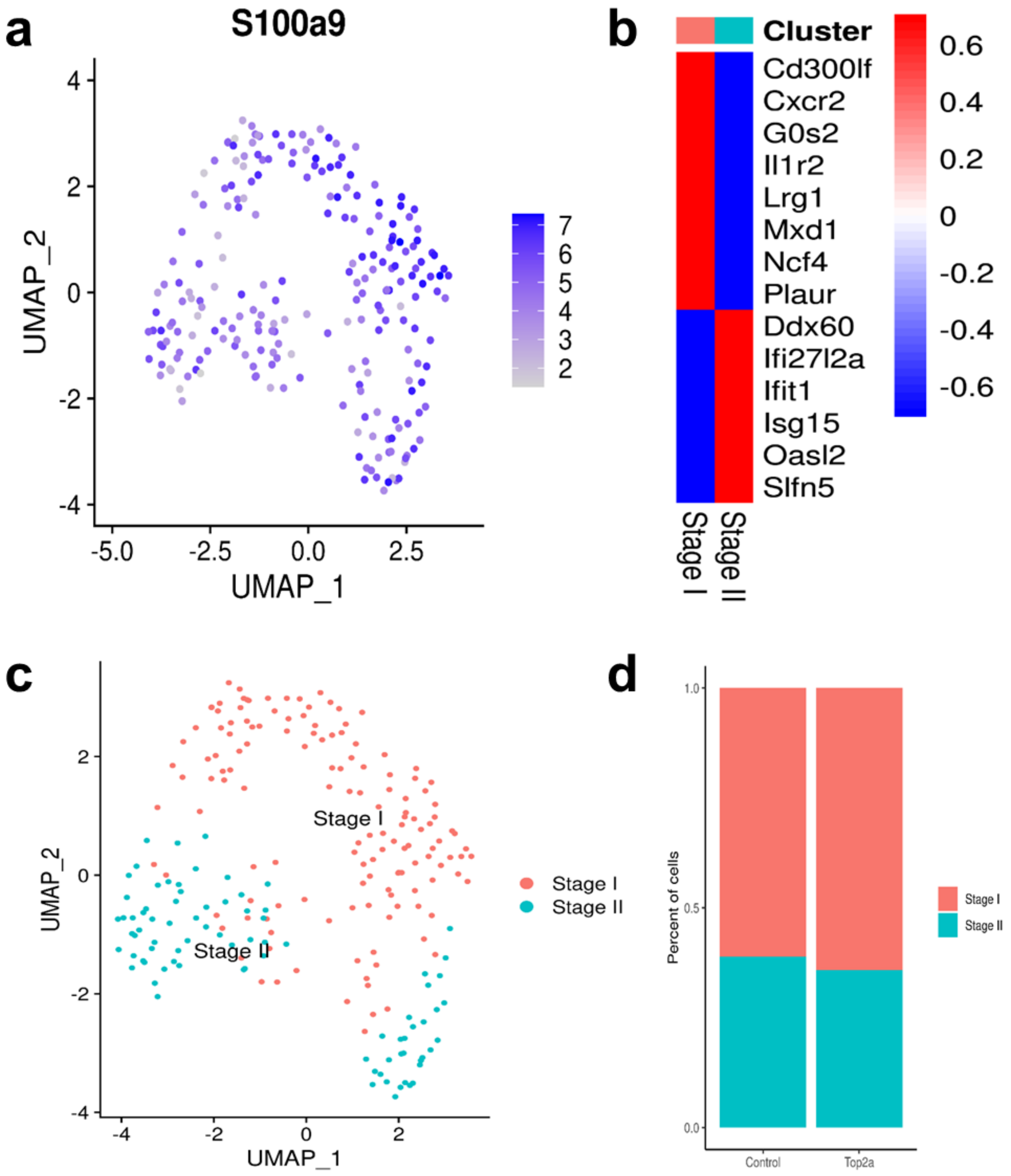

Supplemental Fig. 10 Effects of TOP2A vaccine treatment on neutrophils in breast tumor samples. a Canonical markers expressed on neutrophils were used to generate UMAP plots based on scRNA-seq data. **b** Heatmap based on the expression of the markers for the neutrophil subsets, Stage I and Stage II. **c** UMAP plot distribution of Stage I and Stage II neutrophil subsets. **d** Percent changes in Stage I and Stage II subsets in control versus TOP2A vaccine-treated mice.

Supplemental Figure. 11

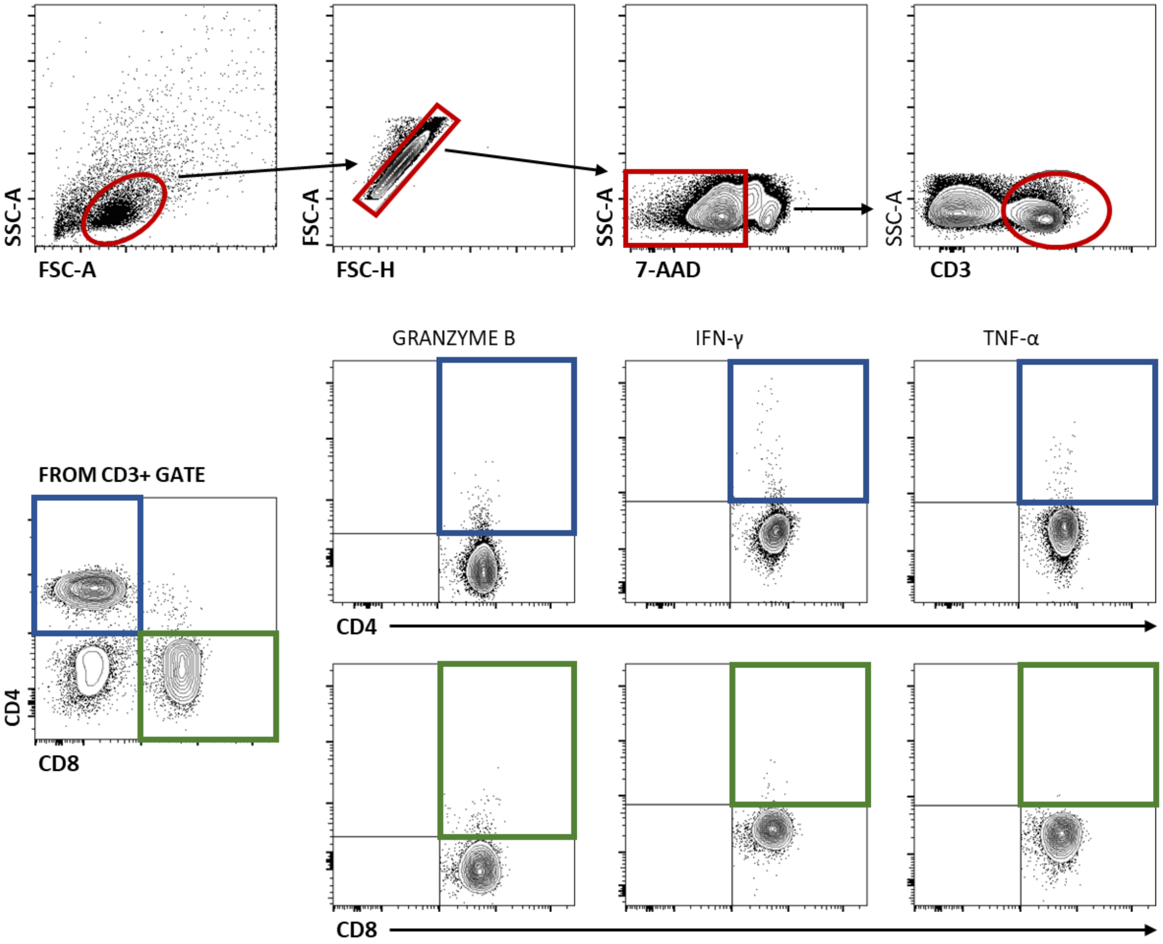

Supplemental Fig. 11 Flow Gating Strategy used for flow cytometry in Figure 2d – i.
