# Supplementary material for: Striking efficacy of a vaccine targeting TOP2A for triple-negative breast cancer immunoprevention
Source: NPJ Precis Oncol. 2023 Oct 25;7:108. doi: 10.1038/s41698-023-00461-1 (PMC10600249; doi:10.1038/s41698-023-00461-1)
Supplement: Supplementary file 3 — REPORTING SUMMARY [file 41698_2023_461_MOESM3_ESM.pdf]

nature portfolio

Corresponding author(s): Yian Wang

Last updated by author(s): Aug 2, 2023

Reporting Summary

Nature Portfolio wishes to improve the reproducibility of the work that we publish. This form provides structure for consistency and transparency in reporting. For further information on Nature Portfolio policies, see our Editorial Policies and the Editorial Policy Checklist.

Statistics

For all statistical analyses, confirm that the following items are present in the figure legend, table legend, main text, or Methods section.

n/a	Confirmed	
☐	☒	The exact sample size (n) for each experimental group/condition, given as a discrete number and unit of measurement
☐	☒	A statement on whether measurements were taken from distinct samples or whether the same sample was measured repeatedly
☐	☒	The statistical test(s) used AND whether they are one- or two-sided<br>*Only common tests should be described solely by name; describe more complex techniques in the Methods section.*
☒	☐	A description of all covariates tested
☒	☐	A description of any assumptions or corrections, such as tests of normality and adjustment for multiple comparisons
☐	☒	A full description of the statistical parameters including central tendency (e.g. means) or other basic estimates (e.g. regression coefficient) AND variation (e.g. standard deviation) or associated estimates of uncertainty (e.g. confidence intervals)
☐	☒	For null hypothesis testing, the test statistic (e.g. F, t, r) with confidence intervals, effect sizes, degrees of freedom and P value noted<br>*Give P values as exact values whenever suitable.*
☒	☐	For Bayesian analysis, information on the choice of priors and Markov chain Monte Carlo settings
☒	☐	For hierarchical and complex designs, identification of the appropriate level for tests and full reporting of outcomes
☒	☐	Estimates of effect sizes (e.g. Cohen's d, Pearson's r), indicating how they were calculated

Our web collection on statistics for biologists contains articles on many of the points above.

Software and code

Policy information about availability of computer code

Data collection	ImmunoSpot Analyzers(CTL Technologies), BD FACSDiva software(BD Biosciences), iMark Microplate Reader(BIO-RAD), NanoZoomer system (Hamamatsu Photonics, Hamamatsu)
Data analysis	GraphPad Prism, FlowJo, NPD.view2(Hamamatsu Photonics, Hamamatsu)

For manuscripts utilizing custom algorithms or software that are central to the research but not yet described in published literature, software must be made available to editors and reviewers. We strongly encourage code deposition in a community repository (e.g. GitHub). See the Nature Portfolio guidelines for submitting code & software for further information.

Data

Policy information about availability of data

All manuscripts must include a data availability statement. This statement should provide the following information, where applicable:

- Accession codes, unique identifiers, or web links for publicly available datasets
- A description of any restrictions on data availability
- For clinical datasets or third party data, please ensure that the statement adheres to our policy

All data are provided in manuscript or Supplementary Information. The raw sequence data generated in this paper have been deposited into the NCBI Sequence Read Archive (SRA) under BioProject accession number PRJNA1001015.

Research involving human participants, their data, or biological material

Policy information about studies with human participants or human data. See also policy information about sex, gender (identity/presentation), and sexual orientation and race, ethnicity and racism.

Reporting on sex and gender	N/A
Reporting on race, ethnicity, or other socially relevant groupings	N/A
Population characteristics	N/A
Recruitment	N/A
Ethics oversight	N/A

Note that full information on the approval of the study protocol must also be provided in the manuscript.

Field-specific reporting

Please select the one below that is the best fit for your research. If you are not sure, read the appropriate sections before making your selection.

☒ Life sciences ☐ Behavioural & social sciences ☐ Ecological, evolutionary & environmental sciences

For a reference copy of the document with all sections, see nature.com/documents/nr-reporting-summary-flat.pdf

Life sciences study design

All studies must disclose on these points even when the disclosure is negative.

Sample size	Sample sizes were determined based on pilot experiments and related literatures (Cohen, J Statistical power analysis for the behavioral sciences (2nd ed.) Hillsdale, NJ: Lawrence Erlbaum).
Data exclusions	No data were excluded for data analyses.
Replication	We used in vivo model, no replication.
Randomization	All animals were randomly assigned to the experimental groups.
Blinding	The researcher were not blinded to experimental group assignments since they need to vaccinate the specific peptide vaccine.

Reporting for specific materials, systems and methods

We require information from authors about some types of materials, experimental systems and methods used in many studies. Here, indicate whether each material, system or method listed is relevant to your study. If you are not sure if a list item applies to your research, read the appropriate section before selecting a response.

Materials & experimental systems

n/a	Involved in the study	
☐	☒	Antibodies
☐	☒	Eukaryotic cell lines
☒	☐	Palaeontology and archaeology
☐	☒	Animals and other organisms
☒	☐	Clinical data
☒	☐	Dual use research of concern
☒	☐	Plants

Methods

n/a	Involved in the study	
☒	☐	ChIP-seq
☐	☒	Flow cytometry
☒	☐	MRI-based neuroimaging

Antibodies

Antibodies used	IHC staining : CD4(14-9766-82, invitrogen), CD8(14-0808-82, invitrogen)<br>Flow cytometry : CD45(304047, Bioledend), CD3(47-0032-82, invitrogen), CD4(116004, Biolegend), CD8(363-0081-82, invitrogen), granzyme B(372203, Biolegend), IFN-γ(505808, Biolegend), and TNF-a(506323, Biolegend)

Validation

All antibodies were validated for the species and application used as stated in the data sheet for antibodies provided by the manufacture.

Eukaryotic cell lines

Policy information about cell lines and Sex and Gender in Research

Cell line source(s)	M6 cells was provided by Dr. Jeffery E. Green (Holzer, R. et al. Breast Cancer Res Treat. 2003)
Authentication	N/A
Mycoplasma contamination	M6 cells tested negative for mycoplasma contamination.
Commonly misidentified lines (See ICLAC register)	We used the M6 cells as a name according to the published paper (Holzer, R. et al. Breast Cancer Res Treat. 2003).

Animals and other research organisms

Policy information about studies involving animals; ARRIVE guidelines recommended for reporting animal research, and Sex and Gender in Research

Laboratory animals	C3 (1)/Tag mice and C3(1)/Tag-REAR mice were a generous gift from Dr. Jeffery E. (GreenAprelikova, O. et al. . PloS One, 2016).
Wild animals	N/A
Reporting on sex	Our study model targeted triple negative breast tumors, we only used female mice.
Field-collected samples	N/A
Ethics oversight	Mice were maintained and bred in the Biomedical Resource Center at the Medical College of Wisconsin (MCW), Milwaukee, WI and the Houston Methodist Research Institute, Houston, TX. All procedures were approved by the Institutional Animal Care and Use Committee (IACUC).

Note that full information on the approval of the study protocol must also be provided in the manuscript.

Flow Cytometry

Plots

Confirm that:

☒ The axis labels state the marker and fluorochrome used (e.g. CD4-FITC).

☒ The axis scales are clearly visible. Include numbers along axes only for bottom left plot of group (a 'group' is an analysis of identical markers).

☒ All plots are contour plots with outliers or pseudocolor plots.

☒ A numerical value for number of cells or percentage (with statistics) is provided.

Methodology

Sample preparation	Whole spleens from mice were processed through large pore mesh or conical top filters for single cell suspensions. Red blood cells were lysed and final cell suspension was counted with viability assessed. One million viable cells were aliquoted per tube for staining and analysis.
Instrument	BD LSR II Flow Cytometer
Software	Flowjo
Cell population abundance	The relevant cell population of interest were T cells. T cells are abundantly found in normal mouse spleens. For this specific data, the T cell population ranges from 30-45% of the samples
Gating strategy	On the FSC/SSC a preliminary gate was placed on debris free lymphocytes. Next single cells were gated based on FSC-H x FSC-A. From the single cell population live/dead cells were determined by 7AAD, cells positive for 7AAD were excluded from further downstream analysis. Next cells were analyzed for CD45 expression and a gate was placed on the positive expressing population. From the CD45+ cells, CD3+ cells were gated followed by a CD4 by CD8 quadrant gate. Further analysis of Granzyme B, IFN-y, and TNF-a was determined for both the CD4 and CD8 T cell populations.

☐ Tick this box to confirm that a figure exemplifying the gating strategy is provided in the Supplementary Information.
